# Synthesis and biological evaluation of 3-arylbenzofuranone derivatives as potential anti-Alzheimer’s disease agents

**DOI:** 10.1080/14756366.2020.1740694

**Published:** 2020-03-18

**Authors:** Jie Yang, Yinling Yun, Yuhang Miao, Jie Sun, Xiaojing Wang

**Affiliations:** a School of Medicine and Life Sciences, University of Jinan-Shandong Academy of Medical Sciences, Shandong, Jinan, China; b Institute of Materia Medica, Shandong First Medical University and Shandong Academy of Medical Sciences, Shandong, Jinan, China; c Key Laboratory for Biotech-Drugs Ministry of Health, Shandong, Jinan, China; d Key Laboratory for Rare and Uncommon Diseases of Shandong Province, Shandong, Jinan, China

**Keywords:** 3-Arylbenzofuranone derivatives, Alzheimer’s disease, cholinesterase inhibitors, monoamine oxidase inhibitors, antioxidant activity

## Abstract

Multi-target drugs can better address the cascade of events involved in oxidative stress and the reduction in cholinergic transmission that occur in Alzheimer’s disease than cholinesterase inhibitors alone. We synthesised a series of 3-arylbenzofuranone derivatives and evaluated their antioxidant activity, cholinesterase inhibitory activity, and monoamine oxidase inhibitory activity. 3-Arylbenzofuranone compounds exhibit good antioxidant activity as well as selective acetylcholinesterase inhibitory activity. The IC_50_ value of anti-acetylcholinesterase inhibition of Compound **20** (0.089 ± 0.01 μM) is similar to the positive drug donepezil (0.059 ± 0.003 μM). According to the experimental results, Compounds **7**, **13** show a certain effect in the *in vitro* evaluation performed and have the potential as drug candidates for the treatment of Alzheimer’s disease.

## Introduction

Alzheimer’s disease (AD) is a chronic neurodegenerative disorder characterised by the destruction of nerve cells, the rapid deterioration of memory and other important cognitive functions^1^. The two major hallmarks of AD are intracellular neurofibrillary tangles composed of hyperphosphorylated T protein^2^ and senile plaques containing aggregated amyloid *β*-peptide (A*β*)^3^. There are other factors that cause synaptic dysfunction and neurodegeneration, which leads to a decrease in neurotransmitter acetylcholine (ACh) levels^4^, leading to memory and cognitive deficits^5^. Monoamine oxidase (MAO) is one of the several enzymes that contribute to the behavioural and psychological symptoms of dementia in AD^6^. Accumulating evidence indicates a close contact between several enzymes and AD. Cholinesterases (ChEs) and MAOs are closely related to the disease symptomatology and progression.

ChEs, enzymes that terminate cholinergic neurotransmission in the brain, act by catalysing the hydrolysis of ACh, so their inhibition can be used to alleviate memory and cognitive deficits in AD. Two types of ChEs, acetylcholinesterase (AChE) and butyrylcholinesterase (BuChE), are known. The difference between the two types is related to their respective preference for the substrate: the former hydrolyses ACh more rapidly; the latter hydrolyses butyrylcholine more rapidly. Experimental studies have found that the maintenance of AChE/BuChE activity ratio in the healthy brain can improve the symptoms of AD^7^. Inhibition of AChE can result in an increase in BuChE activity, which causes hydrolysis of AChE in a novel manner. Therefore, a double ChE inhibitor may be a more effective anti-AD drug. MAO has a major role in brain development and function, and its inhibitors have found clinically as antidepressant and anti-Parkinsonian drugs^6^. MAO regulates the concentration of exogenous and endogenous amines (including neurotransmitters) in the central nervous system and peripheral tissues, which exacerbates subsequent oxidative stress and neurodegeneration.

Benzofuranone and its derivatives are important heterocyclic compounds and are widely used in pesticides, dyes, foods, polymer processing, and other fields. Benzofuranone and its derivatives have rich biological activities, including anticholinesterase^8^, antibacterial^9^, anti-HIV^10^, anti-allergic and anti-inflammatory activity^11^, antioxidant, antinociceptive activity^12^, and other activity, it can be used as a substrate for β-lactamase^13^ and insulin amyloid fibrosis inhibitor^14^ and is also potential antipsychotics^15^. The current study describes the preparation and *in vitro* activity of 3-arylbenzofuranone derivatives as ChE inhibitors, MAO B inhibitors, and 2,2-diphenyl-1-picrylhydrazyl (DPPH) radical scavengers. The purpose of the research is to screen compounds that inhibit ChE and MAO, which have the potential for the treatment of AD and other neurodegenerative diseases.

## Experimental

### Synthesis

#### Materials and methods

Melting points were determined using a Thiele tube and were uncorrected. The ^1^HNMR and 13CNMR spectra were recorded with a Bruker AM-600 spectrometer (Billercia, MA, USA) with TMS as the internal standard. Chemical shifts were reported at room temperature on a scale (ppm) with DMSO-d_6_ as the solvents and *J* values are given in Hertz. Mass spectra were obtained with an Agilent Trap VL LC/MS spectrometer (Santa Clara, CA, USA). The absorbance was recorded by RZ-9618 Microplate Reader. Unless otherwise noted, all solvents and reagents were commercially available and used without further purification.

#### General method for synthesis of compounds 3a-3d

Taking the synthesis of 3, 4, 5-trimethoxy mandelic acid as an example. Other mandelic acids were obtained using the same procedures. 3,4,5-Trimethoxybenzaldehyde 39.2 g (0.2 mol), TBAB 3.2 g (10 mmol), and chloroform 240 ml were added to a 500 ml three-necked flask equipped with a dropping funnel and a reflux condenser. The mixture was thoroughly stirred to completely dissolve, and the temperature was raised to 40 °C. A 50% NaOH solution (40 g of NaOH dissolved in 40 g of water) was slowly added dropwise through a dropping funnel to maintain a temperature of 45–50 °C. After the TLC detection reaction was completed, it was allowed to stand for cooling and suction filtration. The filter cake was washed with chloroform 40 ml × 3. The resulting solid mixture was acidified with hydrochloric acid, extracted with ethyl acetate, dried over anhydrous sodium sulphate, concentrated, and recrystallisation from ethyl acetate/petroleum ether gave a white solid (yield: 70.5%).

#### General method for the synthesis of 3-arylbenzofuranone 1–23

Taking the synthesis of 6-hydroxy-3–(3′,4′,5′-trimethoxyphenyl)-benzofuranone as an example. Other 3-arylbenzofuranone compounds were obtained using the same procedures. 3,4,5-Trimethoxymandelic acid 4.84 g (20 mmol), resorcin 2.64 g (24 mmol), and boron trifluoride-diethyl ether 20 ml were added to a 100 ml three-necked flask equipped with a reflux condenser and a drying tube. The raw material was stirred well to completely dissolve, and maintain the temperature at 30–35 °C continuous stirring. After the TLC detection reaction was completed, the reaction was allowed to stand for cooling. The reaction solution was poured into a beaker containing 100 ml of ice water and thoroughly stirred. After a large amount of white solid was precipitated, it was allowed to stand, and suction filtered. The filter cake was washed with saturated sodium bicarbonate solution, then washed with distilled water until near neutral, dried to give a pale pink solid, and recrystallised from methanol to yield white solid (yield: 92.72%).

6-Hydroxyl-3–(4′-methoxyphenyl)-benzofuranone (**1**). White solid, yield 94.14%, m.p. 188–190 °C. ^1^H NMR (600 MHz, DMSO-d_6_) δ 9.88 (s, 1H), 7.11 (s, 2H), 7.02 − 6.86 (m, 3H), 6.63 (d, *J* = 46.1 Hz, 2H), 5.13 (s, 1H), 3.74 (s, 3H). 13C NMR (151 MHz, DMSO-d_6_) δ 176.19 (s), 158.82 (s), 158.30 (s), 154.05 (s), 129.30 (s), 128.36 (s), 125.61 (s), 117.82 (s), 114.38 (s), 111.32 (s), 98.24 (s), 55.14 (s), 47.84 (s), 39.94 (s), 39.80 (s), 39.66 (s), 39.60 − 39.11 (m), 39.10 (s), 39.09 − 38.90 (m). MS: *m/z* (%) [M + Na]^+^ 278.9.

5-Hydroxyl-3–(4′-methoxyphenyl)-benzofuranone (**2**). White solid, yield 95.12%, m.p. 164–165 °C. ^1^H NMR (600 MHz, DMSO-d_6_) δ 9.38 (s, 1H), 7.13 − 7.08 (m, 3H), 6.96 − 6.93 (m, 2H), 6.74 (ddd, *J* = 8.7, 2.6, 0.7 Hz, 1H), 6.55 (dd, *J* = 2.5, 0.9 Hz, 1H), 5.23 (s, 1H), 3.75 (s, 3H). 13C NMR (151 MHz, DMSO-d_6_) δ 176.09 (s), 158.85 (s), 154.32 (s), 145.74 (s), 129.40 (s), 128.99 (s), 127.94 (s), 115.10 (s), 114.44 (s), 111.74 (s), 111.02 (s), 55.15 (s), 48.89 (s), 40.02 (d, *J* = 11.3 Hz), 39.98 − 39.62 (m), 39.55 (s), 39.52 (s), 39.38 (s), 39.29 (s), 39.17 (d, *J* = 21.0 Hz). MS: *m/z* (%) [M + Na]^+^ 279.0.

6-Methoxy-3–(4′-methoxyphenyl)-benzofuranone (**3**). White solid, yield 85.32%, m.p. 156–157 °C. ^1^H NMR (600 MHz, DMSO-d_6_) δ 7.10 (dd, *J* = 11.4, 4.6 Hz, 3H), 6.96 − 6.92 (m, 3H), 6.75 (dd, *J* = 8.3, 2.3 Hz, 1H), 5.20 (s, 1H), 3.79 (s, 3H), 3.74 (s, 3H). 13C NMR (151 MHz, DMSO-d_6_) δ 176.03 (s), 160.14 (s), 158.87 (s), 154.16 (s), 129.33 (s), 128.13 (s), 125.62 (s), 119.61 (s), 114.42 (s), 110.19 (s), 97.22 (s), 55.66 (s), 55.15 (s), 47.86 (s), 39.87 (d, *J* = 21.0 Hz), 39.66 (s), 39.52 (s), 39.38 (s), 39.24 (s), 39.10 (s). MS: *m/z* (%) [M + Na]^+^ 293.0, [M + H]^+^ 271.0.

5-Methoxy-3–(4′-methoxyphenyl)-benzofuranone (**4**). White solid, yield 76.32%, m.p. 126–127 °C. ^1^H NMR (600 MHz, DMSO-d_6_) δ 7.23 (d, *J* = 8.8 Hz, 1H), 7.12 (d, *J* = 8.7 Hz, 2H), 6.97 − 6.92 (m, 3H), 6.79 − 6.74 (m, 1H), 5.28 (s, 1H), 3.75 (s, 3H), 3.70 (s, 3H). 13C NMR (151 MHz, DMSO-d_6_) δ 175.92 (s), 158.90 (s), 156.37 (s), 146.97 (s), 129.46 (s), 129.13 (s), 127.74 (s), 114.47 (s), 114.22 (s), 111.11 (s), 110.70 (s), 55.68 (s), 55.15 (s), 48.95 (s), 39.94 (s), 39.73 (d, *J* = 21.0 Hz), 39.52 (s), 39.39 (s), 39.38 (s), 39.24 (s), 39.10 (s). MS: *m/z* (%) [M + Na]^+^ 293.0, [M + H]^+^ 271.0.

6,7-Dihydroxy-3–(4′-methoxyphenyl)-benzofuranone (**5**). White solid, yield 91.91%, m.p. 138–140 °C. ^1^H NMR (600 MHz, DMSO-d_6_) δ 7.12 − 7.09 (m, 2H), 6.94 − 6.91 (m, 2H), 6.59 (d, *J* = 8.0 Hz, 1H), 6.43 (dd, *J* = 8.0, 1.1 Hz, 1H), 5.15 (s, 1H), 3.74 (s, 3H). 13C NMR (151 MHz, DMSO-d_6_) δ 176.16 (s), 158.81 (s), 146.82 (s), 141.77 (s), 129.93 (s), 129.35 (s), 128.56 (s), 119.11 (s), 114.67 (s), 114.39 (s), 111.45 (s), 55.18 (s), 48.53 (s), 39.94 (s), 39.81 (s), 39.80 − 39.52 (m), 39.39 (s), 39.31 (d, *J* = 21.0 Hz), 39.10 (s). MS: *m/z* (%) [M + Na]^+^ 295.0, [M + H]^+^ 273.0.

6-Hydroxy-3–(3′,4′-dimethoxyphenyl)-benzofuranone (**6**). White solid, yield 60.47%, m.p. 171–172 °C. ^1^H NMR (600 MHz, DMSO-d_6_) δ 9.88 (s, 1H), 7.00 (dd, *J* = 8.2, 0.9 Hz, 1H), 6.93 (d, *J* = 8.3 Hz, 1H), 6.81 (d, *J* = 2.0 Hz, 1H), 6.67 (d, *J* = 2.2 Hz, 1H), 6.64 (dd, *J* = 8.3, 2.0 Hz, 1H), 6.59 (dd, *J* = 8.2, 2.2 Hz, 1H), 5.12 (s, 1H), 3.73 (s, 3H), 3.71 (s, 3H). 13C NMR (151 MHz, DMSO-d_6_) δ 158.29 (s), 154.03 (s), 148.95 (s), 148.44 (s), 128.68 (s), 125.64 (s), 120.14 (s), 117.76 (s), 112.08 (d, *J* = 15.6 Hz), 111.31 (s), 98.25 (s), 55.54 (d, *J* = 3.3 Hz), 48.19 (s), 39.87 (d, *J* = 20.9 Hz), 39.67 (s), 39.59 (d, *J* = 21.0 Hz), 39.41 (s), 39.38 (s), 39.17 (d, *J* = 21.0 Hz). MS: *m/z* (%) [M + Na]^+^ 308.9.

5-Hydroxy-6-methoxy-3–(4′-methoxyphenyl)-benzofuranone (**7**). White solid, yield 83.92%, m.p. 151–153 °C. ^1^H NMR (600 MHz, DMSO-d_6_) δ 8.92 (s, 1H), 7.11 − 7.08 (m, 2H), 7.00 (s, 1H), 6.95 − 6.92 (m, 2H), 6.57 (d, *J* = 0.7 Hz, 1H), 5.14 (s, 1H), 3.81 (s, 3H), 3.74 (s, 3H). 13C NMR (151 MHz, DMSO-d_6_) δ 176.46 (s), 158.82 (s), 148.21 (s), 145.91 (s), 143.58 (s), 129.30 (s), 128.29 (s), 118.50 (s), 114.39 (s), 111.34 (s), 96.47 (s), 56.13 (s), 55.15 (s), 48.54 (s), 40.00 (d, *J* = 16.4 Hz), 39.87 (d, *J* = 21.0 Hz), 39.66 (s), 39.52 (s), 39.38 (s), 39.24 (s), 39.10 (s). MS: *m/z* (%) [M + Na]^+^ 309.0.

6-Methoxy-7-hydroxy-3–(4′-methoxyphenyl)-benzofuranone (**8**). White solid, yield 86.36%, m.p. 138–139 °C. ^1^H NMR (600 MHz, DMSO-d_6_) δ 9.44 (s, 1H), 7.11 (d, *J* = 8.5 Hz, 2H), 6.93 (d, *J* = 8.6 Hz, 2H), 6.77 (d, *J* = 8.2 Hz, 1H), 6.57 (d, *J* = 8.2 Hz, 1H), 5.20 (s, 1H), 3.80 (s, 3H), 3.74 (s, 3H). 13C NMR (151 MHz, DMSO-d_6_) δ 175.87 (s), 158.82 (s), 148.87 (s), 141.31 (s), 130.99 (s), 129.37 (s), 128.31 (s), 121.27 (s), 114.43 (d, *J* = 13.2 Hz), 108.07 (s), 56.27 (s), 55.16 (s), 48.44 (s), 39.94 (s), 39.80 (s), 39.66 (s), 39.52 (s), 39.38 (s), 39.24 (s), 39.10 (s). MS: *m/z* (%) [M + Na]^+^ 309.0, [M + H]^+^ 287.0.

6-Methoxy-3–(3′,4′-dimethoxyphenyl)-benzofuranone (**9**). White solid, yield 62.81%, m.p. 125–126 °C. ^1^H NMR (600 MHz, DMSO-d_6_) δ 7.12 (d, *J* = 7.7 Hz, 1H), 6.99 − 6.89 (m, 2H), 6.83 (s, 1H), 6.75 (d, *J* = 7.4 Hz, 1H), 6.65 (d, *J* = 7.0 Hz, 1H), 5.18 (s, 1H), 3.80 (s, 3H), 3.76 − 3.68 (m, 6H). 13C NMR (151 MHz, DMSO-d_6_) δ 175.87 (s), 160.11 (s), 154.13 (s), 148.73 (d, *J* = 72.9 Hz), 128.42 (s), 125.62 (s), 120.16 (s), 119.53 (s), 112.10 (d, *J* = 11.7 Hz), 110.16 (s), 97.20 (s), 55.59 (d, *J* = 16.4 Hz), 48.19 (s), 39.97 − 39.30 (m), 39.17 (d, *J* = 21.0 Hz). MS: *m/z* (%) [M + Na]^+^ 323.0, [M + H]^+^ 301.1, [2M + Na]^+^ 623.1.

5,6-Dimethoxy-3–(4′-methoxyphenyl)-benzofuranone (**10**). White solid, yield 91.11%, m.p. 128–130 °C. ^1^H NMR (600 MHz, DMSO-d_6_) δ 7.12 (d, *J* = 8.7 Hz, 2H), 7.07 (s, 1H), 6.94 (d, *J* = 8.8 Hz, 2H), 6.79 (d, *J* = 0.5 Hz, 1H), 5.18 (s, 1H), 3.81 (s, 3H), 3.75 (s, 3H), 3.67 (s, 3H). 13C NMR (151 MHz, DMSO-d_6_) δ 176.34 (s), 158.87 (s), 149.74 (s), 147.12 (s), 146.14 (s), 129.38 (s), 128.13 (s), 117.96 (s), 114.43 (s), 108.67 (s), 96.44 (s), 56.28 (s), 56.09 (s), 55.15 (s), 48.66 (d, *J* = 15.2 Hz), 40.00 (d, *J* = 18.1 Hz), 39.80 (s), 39.59 (d, *J* = 21.0 Hz), 39.43 (s), 39.38 (s), 39.24 (s), 39.10 (s). MS: *m/z* (%) [M + Na]^+^ 323.0, [M + H]^+^ 301.1, [2M + Na]^+^ 623.1.

6-Hydroxy-3–(3′,4′,5′-trimethoxyphenyl)-benzofuranone (**11**). White solid, yield 92.72%, m.p. 191–193 °C. ^1^H NMR (600 MHz, DMSO-d_6_) δ 9.90 (s, 1H), 7.04 (dd, *J* = 8.2, 1.0 Hz, 1H), 6.67 (d, *J* = 2.2 Hz, 1H), 6.60 (dd, *J* = 8.2, 2.2 Hz, 1H), 6.47 (s, 2H), 5.14 (s, 1H), 3.72 (s, 6H), 3.65 (s, 3H). 13C NMR (151 MHz, DMSO-d_6_) δ 175.71 (s), 158.35 (s), 154.04 (s), 153.17 (s), 137.05 (s), 131.93 (s), 125.68 (s), 117.42 (s), 111.35 (s), 105.53 (s), 98.31 (s), 60.00 (s), 55.92 (s), 48.76 (s), 39.94 (s), 39.80 (s), 39.66 (s), 39.52 (s), 39.31 (d, *J* = 21.0 Hz), 39.24 − 39.23 (m), 39.10 (s). MS: *m/z* (%)[M + Na]^+^ 339.3.

6-Methoxy-7-hydroxy-3–(3′,4′-dimethoxyphenyl)-benzofuranone (**12**). White solid, yield 78.13%, m.p. 135–137 °C. ^1^H NMR (600 MHz, DMSO-d_6_) δ 9.43 (s, 1H), 6.93 (d, *J* = 8.3 Hz, 1H), 6.82 (d, *J* = 1.9 Hz, 1H), 6.77 (d, *J* = 8.3 Hz, 1H), 6.66 (dd, *J* = 8.3, 1.9 Hz, 1H), 6.60 (d, *J* = 8.2 Hz, 1H), 5.18 (s, 1H), 3.81 (s, 3H), 3.74 (s, 3H), 3.71 (s, 3H). 13C NMR (151 MHz, DMSO-d_6_) δ 175.72 (s), 148.91 (d, *J* = 13.3 Hz), 148.45 (s), 141.30 (s), 130.95 (s), 128.60 (s), 121.17 (s), 120.25 (s), 114.49 (s), 112.10 (d, *J* = 8.6 Hz), 108.00 (s), 56.25 (s), 55.55 (s), 48.78 (s), 48.62 (s), 39.87 (d, *J* = 21.0 Hz), 39.66 (s), 39.64 − 39.62 (m), 39.52 (s), 39.38 (s), 39.24 (s), 39.10 (s). MS: *m/z* (%) [M + Na]^+^ 339.0, [M + H]^+^ 317.1, [2M + Na]^+^ 655.1.

5-Hydroxy-6-methoxy-3–(3′,4′-dimethoxyphenyl)-benzofuranone (**13**). White solid, yield 79.11%, m.p. 154–155 °C. ^1^H NMR (600 MHz, DMSO-d_6_) δ 8.91 (d, *J* = 1.5 Hz, 1H), 7.00 (d, *J* = 1.8 Hz, 1H), 6.93 (dd, *J* = 8.3, 1.8 Hz, 1H), 6.81 (s, 1H), 6.63 (d, *J* = 8.2 Hz, 1H), 6.59 (s, 1H), 5.12 (s, 1H), 3.81 (d, *J* = 1.8 Hz, 3H), 3.73 (dd, *J* = 10.9, 1.8 Hz, 6H). 13C NMR (151 MHz, DMSO-d_6_) δ 176.31 (s), 148.96 (s), 148.45 (s), 148.19 (s), 145.89 (s), 143.53 (s), 128.61 (s), 120.13 (s), 118.43 (s), 112.10 (d, *J* = 13.5 Hz), 111.35 (s), 96.43 (s), 56.11 (s), 55.55 (s), 48.88 (s), 39.94 (s), 39.90 − 39.44 (m), 39.38 (s), 39.24 (s), 39.10 (s). MS: *m/z* (%) [M + Na]^+^ 339.0, [2M + Na]^+^ 655.1.

5-Hydroxy-3–(3′,4′,5′-trimethoxyphenyl)-benzofuranone (**14**). White solid, yield 50.68%, m.p. 188–189 °C. ^1^H NMR (600 MHz, DMSO-d_6_) δ 9.38 (s, 1H), 7.10 (d, *J* = 8.7 Hz, 1H), 6.75 (ddd, *J* = 8.7, 2.6, 0.7 Hz, 1H), 6.61 (dd, *J* = 2.5, 0.9 Hz, 1H), 6.49 (s, 2H), 5.23 (s, 1H), 3.73 (s, 6H), 3.66 (s, 3H). 13C NMR (151 MHz, DMSO-d_6_) δ 175.61 (s), 154.29 (s), 153.21 (s), 145.70 (s), 137.14 (s), 131.52 (s), 128.65 (s), 115.23 (s), 111.74 (s), 111.08 (s), 105.67 (s), 60.02 (s), 55.97 (s), 49.83 (s), 39.87 (d, *J* = 21.0 Hz), 39.68 (s), 39.66 (s), 39.52 (s), 39.38 (s), 39.17 (d, *J* = 21.0 Hz). MS: *m/z* (%) [M + H]^+^ 317.1, [M + Na]^+^ 339.1, [2M + Na]^+^ 655.2.

5-Methoxy-3–(3′,4′,5′-trimethoxyphenyl)-benzofuranone (**15**). White solid, yield 78.79%, m.p. 141–142 °C. ^1^H NMR (600 MHz, DMSO-d_6_) δ 7.23 (d, *J* = 8.8 Hz, 1H), 6.95 (ddd, *J* = 8.8, 2.7, 0.7 Hz, 1H), 6.84 (dd, *J* = 2.7, 1.0 Hz, 1H), 6.49 (s, 2H), 5.28 (s, 1H), 3.73 (s, 6H), 3.72 (s, 3H), 3.66 (s, 3H). 13C NMR (151 MHz, DMSO-d_6_) δ 175.47 (s), 156.38 (s), 153.23 (s), 146.95 (s), 137.16 (s), 131.26 (s), 128.71 (s), 114.43 (s), 111.18 (s), 110.71 (s), 105.72 (s), 60.01 (s), 55.97 (s), 55.76 (s), 49.89 (s), 39.94 (s), 39.80 (s), 39.66 (s), 39.52 (s), 39.38 (s), 39.24 (s), 39.10 (s). MS: *m/z* (%) [M + H]^+^ 330.9.

6-Methoxy-3–(3′,4′,5′-trimethoxyphenyl)-benzofuranone (**16**). White solid, yield 90.16%, m.p. 153–154 °C. ^1^H NMR (600 MHz, DMSO-d_6_) δ 7.17 (d, *J* = 8.4 Hz, 1H), 6.96 (d, *J* = 2.2 Hz, 1H), 6.76 (dd, *J* = 8.3, 2.2 Hz, 1H), 6.49 (s, 2H), 5.20 (s, 1H), 3.80 (s, 3H), 3.72 (s, 6H), 3.65 (s, 3H). 13C NMR (151 MHz, DMSO-d_6_) δ 175.54 (s), 160.18 (s), 154.15 (s), 153.20 (s), 137.12 (s), 131.66 (s), 125.67 (s), 119.18 (s), 110.19 (s), 105.59 (s), 97.24 (s), 59.99 (s), 55.93 (s), 55.65 (s), 48.78 (s), 39.94 (s), 39.73 (d, *J* = 21.0 Hz), 39.57 (s), 39.52 (s), 39.38 (s), 39.24 (s), 39.10 (s). MS: *m/z* (%) [M + H]^+^ 331.1, [M + Na]^+^ 353.1, [2M + Na]^+^ 683.2.

5-Methoxy-3–(2′,3′,4′-trimethoxyphenyl)-benzofuranone (**17**). White solid, yield 84.85%, m.p. 132–135 °C. ^1^H NMR (600 MHz, DMSO-d_6_) δ 7.17 (d, *J* = 8.7 Hz, 1H), 7.13 (d, *J* = 8.4 Hz, 1H), 6.89 − 6.86 (m, 1H), 6.84 (d, *J* = 8.6 Hz, 1H), 6.59 − 6.57 (m, 1H), 5.14 (s, 1H), 3.81 (s, 3H), 3.69 (s, 3H), 3.67 (s, 3H). 13C NMR (151 MHz, DMSO-d_6_) δ 176.56 (s), 156.57 (s), 154.30 (s), 147.54 (s), 142.16 (s), 130.53 (s), 125.91 (s), 122.95 (s), 114.01 (s), 111.07 (s), 110.34 (s), 108.04 (s), 60.69 (s), 60.42 (s), 56.36 (s), 56.11 (s), 43.89 (d, *J* = 1014.6 Hz), 40.52 (s), 40.57 − 40.18 (m), 40.18 − 39.79 (m), 39.81 − 39.79 (m), 39.69 (s), 39.55 (s). MS: *m/z* (%) [M + Na]^+^ 353.1, [2M + Na]^+^ 683.2.

6-Methoxy-3–(2′,3′,4′-trimethoxyphenyl)-benzofuranone (**18**). White solid, yield 75.76%, m.p. 100–101 °C. ^1^H NMR (600 MHz, DMSO-d_6_) δ 7.12 (d, *J* = 8.5 Hz, 1H), 6.92 (dd, *J* = 9.6, 1.6 Hz, 2H), 6.83 (d, *J* = 8.6 Hz, 1H), 6.66 (dd, *J* = 8.3, 2.4 Hz, 1H), 5.06 (s, 1H), 3.81 (s, 3H), 3.77 (s, 3H), 3.69 (s, 3H), 3.33 (s, 3H). 13C NMR (151 MHz, DMSO-d_6_) δ 176.68 (s), 160.31 (s), 154.61 (s), 154.22 (s), 151.07 (s), 142.21 (s), 125.71 (s), 124.98 (s), 123.39 (s), 121.12 (s), 109.91 (s), 108.05 (s), 97.47 (s), 60.68 (s), 60.44 (s), 56.36 (s), 56.04 (s), 46.15 (s), 40.33 (d, *J* = 21.0 Hz), 40.12 (s), 39.98 (s), 39.84 (s), 39.70 (s), 39.56 (s). MS: *m/z* (%) [M + Na]^+^ 353.1, [2M + Na]^+^ 683.2.

5,6-Dimethoxy-3–(3′,4′-dimethoxyphenyl)-benzofuranone (**19**). White solid, yield 84.85%, m.p. 149–151 °C. ^1^H NMR (600 MHz, DMSO-d_6_) δ 7.06 (s, 1H), 6.94 (d, *J* = 8.3 Hz, 1H), 6.83 − 6.80 (m, 2H), 6.66 (d, *J* = 8.2 Hz, 1H), 5.17 (s, 1H), 3.81 (s, 3H), 3.74 (s, 3H), 3.72 (s, 3H), 3.68 (s, 3H). 13C NMR (151 MHz, DMSO-d_6_) δ 176.18 (s), 149.71 (s), 148.95 (s), 148.49 (s), 147.10 (s), 146.09 (s), 128.39 (s), 120.24 (s), 117.85 (s), 112.11 (d, *J* = 11.0 Hz), 108.68 (s), 96.40 (s), 56.29 (s), 56.06 (s), 55.53 (s), 54.91 (s), 49.03 (s), 39.87 (d, *J* = 21.0 Hz), 39.66 (s), 39.52 (s), 39.52 (s), 39.38 (s), 39.24 (s), 39.10 (s). MS: *m/z* (%) [M + Na]^+^ 353.1, [2M + Na]^+^ 683.2.

6,7-Dihydroxy−3–(3′,4′,5′-trimethoxyphenyl)-benzofuranone (**20**). White solid, yield 87.65%, m.p. 170–172 °C. ^1^H NMR (600 MHz, DMSO-d_6_) δ 9.49 (s, 1H), 9.26 (s, 1H), 6.60 (d, *J* = 8.1 Hz, 1H), 6.50 (dd, *J* = 8.1, 1.1 Hz, 1H), 6.47 (s, 2H), 5.15 (s, 1H), 3.72 (s, 6H), 3.65 (s, 3H). 13C NMR (151 MHz, DMSO-d_6_) δ 175.61 (s), 153.15 (s), 146.86 (s), 141.74 (s), 132.06 (s), 129.89 (s), 118.62 (s), 114.72 (s), 111.45 (s), 105.53 (s), 60.01 (s), 55.91 (s), 49.41 (s), 39.87 (d, *J* = 21.0 Hz), 39.76 − 39.72 (m), 39.66 (s), 39.52 (s), 39.38 (s), 39.24 (s), 39.10 (s). MS: *m/z* (%) [M + H]^+^ 332.9.

5-Hydroxy-6-methoxy-3–(3′,4′,5′-trimethoxyphenyl)-benzofuranone (**21**). White solid, yield 83.24%, m.p. 178–180 °C. ^1^H NMR (600 MHz, DMSO-d_6_) δ 8.92 (s, 1H), 7.00 (s, 1H), 6.63 (d, *J* = 0.9 Hz, 1H), 6.47 (s, 2H), 5.14 (s, 1H), 3.81 (s, 3H), 3.72 (s, 6H), 3.66 (s, 3H). 13C NMR (151 MHz, DMSO-d_6_) δ 175.97 (s), 153.19 (s), 148.27 (s), 145.90 (s), 143.52 (s), 137.09 (s), 131.88 (s), 118.11 (s), 111.33 (s), 105.55 (s), 96.44 (s), 60.01 (s), 56.09 (s), 55.95 (s), 49.49 (s), 39.94 (s), 39.73 (d, *J* = 21.0 Hz), 39.52 (s), 39.38 (s), 39.24 (s), 39.10 (s). MS: *m/z* (%) [M + H]^+^ 347.1, [M + Na]^+^ 369.1, [2M + Na]^+^ 715.2.

6-Methoxy-7-hydroxy-3–(3′,4′,5′-trimethoxyphenyl)-benzofuranone (**22**). White solid, yield 76.59%, m.p. 200–202 °C. ^1^H NMR (600 MHz, DMSO-d_6_) δ 9.44 (s, 1H), 6.78 (d, *J* = 8.3 Hz, 1H), 6.64 (dd, *J* = 8.2, 1.1 Hz, 1H), 6.49 (s, 2H), 5.21 (d, *J* = 0.7 Hz, 1H), 3.81 (s, 3H), 3.72 (s, 6H), 3.65 (s, 3H). 13C NMR (151 MHz, DMSO-d_6_) δ 175.83 (s), 153.62 (s), 149.38 (s), 141.75 (s), 137.53 (s), 132.27 (s), 131.39 (s), 121.24 (s), 115.00 (s), 108.41 (s), 106.08 (s), 60.45 (s), 56.67 (s), 56.39 (s), 49.82 (s), 40.39 (s), 40.25 (s), 40.11 (s), 39.90 (d, *J* = 21.0 Hz), 39.69 (s), 39.55 (s). MS: *m/z* (%) [M + H]^+^ 347.1044, [M + Na]^+^ 369.0866, [2M + Na]^+^ 715.1836.

5-Hydroxy-6-methoxy-3–(3′,4′,5′-trimethoxyphenyl)-benzofuranone (**23**). White solid, yield 84.26%, m.p. 183–184 °C. ^1^H NMR (600 MHz, DMSO-d_6_) δ 7.07 (s, 1H), 6.86 (d, *J* = 0.6 Hz, 1H), 6.49 (s, 2H), 5.19 (s, 1H), 3.81 (s, 3H), 3.73 (s, 6H), 3.69 (d, *J* = 4.3 Hz, 3H), 3.66 (s, 3H). 13C NMR (151 MHz, DMSO-d_6_) δ 175.83 (s), 153.19 (s), 149.79 (s), 147.15 (s), 146.12 (s), 137.10 (s), 131.57 (s), 117.40 (s), 108.74 (s), 105.61 (s), 96.40 (s), 59.97 (s), 56.34 (s), 55.98 (d, *J* = 18.2 Hz), 49.58 (s), 39.87 (d, *J* = 21.0 Hz), 39.67 (s), 39.59 (d, *J* = 21.0 Hz), 39.38 (s), 39.25 (s), 39.17 (d, *J* = 21.0 Hz). MS: *m/z* (%) [M + H]^+^ 361.1, [M + Na]^+^ 383.1, [2M + Na]^+^ 743.2.

### Biological activity

#### Animals

Wistar rat, weight 200–250 g, were obtained from Jinan Peng Yue Experimental Animal Co. (License number: SCXK (Lu) 2014–0007), Ltd. The animals were housed under standard laboratory conditions and maintained on a standard pellet diet and water *ad libitum*. All experiments involving living animals and their care were performed in strict accordance with the National Care and Use of Laboratory Animals by the National Animal Research Authority (China) and guidelines of Animal Care and Use issued by University of Jinan Institutional Animal Care and Use Committee. The experiments were approved by the Institutional Animal Care and Use Committee of the School of Medicine and Life Sciences, University of Jinan. All efforts were made to minimise animal’s suffering and to reduce the number of animals used.

#### *In vitro* ChE inhibitory activity

The anticholinesterase activity of the 3-arylbenzofuranone compounds was determined by the method of Ellman et al.^16^ with slight modifications. The *in vitro* inhibition assays of AChE from electric eel and BChE from equine serum were run in phosphate buffer 0.1 M, at pH 8.0. Acetylthiocholine iodide and butyrylthiocholine iodide were used as substrates respectively. 5,5′-Dithiobis(2-nitrobenzoic acid) (DTNB) was used as the chromophoric reagent. To a 96-well plate, 120 µL of phosphate buffer solution (0.1 M, pH = 8.0, PBS), 20 µL of DTNB (3.3 mM in 0.1 M PBS, pH = 8.0) were added sequentially, 20 µL AChE solution (0.2 U/mL in 0.1 M PBS, pH = 8.0), 20 µL of different concentrations of the sample solution, shaken well, and incubated at 37 °C for 5 min. Then, 20 µL of substrate (5 mM in 0.1 M PBS, pH = 8.0) were added, shaken well, and incubated at 37 °C for 20 min. The absorbance at 412 nm of the samples was measured using a spectrophotometer, and the inhibition rate of ChE and the IC_50_ value of each sample were calculated according to the formula. BChE inhibitory activity was assessed similarly using butyrylthiocholine iodide as the substrate. The sample solution was set to five concentration gradients and the experiment was repeated three times. Donepezil was used as a positive control.

$$\text{Cholinesterase}\;\text{inhibitory}\;\text{effect}\;\left(\%\right)=\left[A_{0}-\left(A_{1}-A_{2}\right)\right]/A_{0}\times 100\%$$

where *A*_0_ is the absorbance of blank group; *A*_1_ is the absorbance of sample group; *A*_2_ is the absorbance of sample blank group.

#### *In vitro* MAO inhibitory activity

The MAO inhibitory activity of the 3-arylbenzofuranone compounds was determined by the method of Holt et al.^17^ with slight modifications. The crude enzyme was extracted from the liver of 200–250 g of Wistar rats according to literature methods^18^^,^^19^. The crude enzyme protein content was determined by the Bradford method using a Bradford Protein Assay Kit (Beyotime). The assay was performed in 0.2 M potassium phosphate buffer pH 7.6 on 96-well plates in 240 μl total volume. The chromogenic solution containing 1 mM vanillic acid, 0.5 mM 4-aminoantipyrine, and 4 U/ml horseradish peroxidase in 0.2 M potassium phosphate buffer pH 7.6 was mixed anew for each measurement. 40 μL of enzyme solution and 40 μL of sample solution were added to a 96-well plate. The solution was then incubated at 37 °C for 20 min. 120 μL of the 4-(trifluoromethyl) benzylamine solution and 40 μL of the chromogenic solution were added subsequently and incubated at 37 °C for 90 min. The absorbance was measured at 490 nm using a microplate reader, and the inhibition rate of MAO and the IC_50_ value of each sample were calculated according to the formula. The control group replaced the sample solution with PBS (0.2 M, pH = 7.6), the positive control replaced the sample with the positive drug, and the blank group replaced the substrate with PBS, and each group was measured three times in parallel to average.

$$\text{Monoamine oxidase inhibitory effect }(\%)\;=\;[(A_{C}\;–\;A_{B})\text{ – }(A_{S}\;–A_{\mathrm{SB}}\;)]/(A_{C}\;–{\;A}_{B})\times 100\%$$

where *A*_C_ is the absorbance of control group; *A*_B_ is the absorbance of blank group; *A*_S_ is the absorbance of sample group; *A*_SB_ is the absorbance of sample blank group.

#### *In vitro* antioxidant activity

The assay provides an assessment of the antioxidant activity due to free radical scavenging by measuring hydrogen atom (or one electron) donating activity. DPPH is a purple stable free radical that is reduced to a yellow-coloured diphenylpicryl hydrazine by an antioxidant. The spectrophotometric assay was carried out as described by the literature method with a slight modification^20^. 100 μL of a sample solution was added to 100 μL of DPPH solution. Following 30 min of incubation at 37 °C and protection from light, the absorbance at 517 nm was determined using a microplate reader. Each group was measured three times in parallel to average. IC_50_ values were calculated. The percentage of DPPH radical scavenging rate of the target compound is calculated as follows:

$$\text{DPPH}\;\text{free}\;\text{radical}\;\text{scavenging}\;\text{effect}\;\left(\%\right)=\left[A_{0}\;–\;\left(A_{1}-A_{2}\right)\right]/A_{0}\times 100\%$$

where *A*_0_ is the absorbance of blank group; *A*_1_ is the absorbance of sample group; *A*_2_ is the absorbance of sample blank group.

#### Kinetic characterisation of ChEs inhibition

To obtain the mechanism of action of **13**, reciprocal plots of 1/velocity versus 1/[substrate] were constructed at different concentrations of the substrate thiocholine iodide by using Ellman’s method. Compound **13** was added to the assay solution and preincubated with the enzyme at 37 °C for 5 min, followed by the addition of the substrate. Kinetic characterisation catalysed by enzyme was achieved spectrometrically at 412 nm. A parallel control experiment was carried out without compound **13** in the mixture. Lineweaver–Burk plot was made based on the reaction rate and substrate concentration to determine the type of inhibition of the ChE by the compound.

#### Molecular modelling

The docking of the compounds was performed using the Accelrys Discovery Studio 2019 (DS, Accelrys Software Inc., San Diego, CA, USA) CDOCKER docking protocol. A simulation system was constructed based on the structure obtained from the protein database (PDB: 6O4W for AChE; 6EP4 for BuChE; 4A79 for MAO-B). Preparing the protein using prepare protein method building in DS retains water in the protein-binding pocket. The active site is defined by the ligand of the protein crystal structure, and then the original crystal ligand is docked again, and the RMSD value obtained is less than 1. Prior to the docking calculation, the original ligand was removed. The 3D structure of the compound was generated and optimised using DS.

#### TOPKAT prediction

TOPKAT protocol in DS was also used to predict the rat oral LD_50_ and potential toxicity (i.e. carcinogenicity and mutagenicity) of the compounds (Table 3).

#### Statistical analysis

Data were shown as mean ± SD Differences between individual groups were analysed by using ANOVA followed by Dunett’s test. A difference with a *p* values of <0.05 was considered to be significant.

## Results and discussion

### Chemistry

**Scheme 1.**
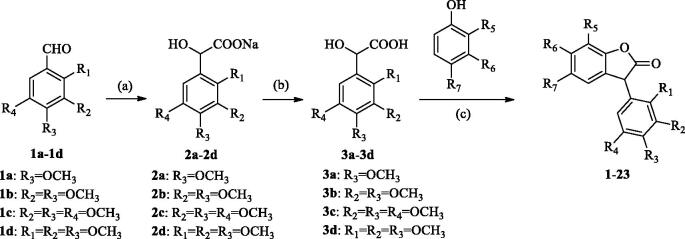
General synthetic route to 3-arylbenzofuranone, reagents and conditions: (a) CHCl_3_, TBAB, NaOH, 40–50 °C; (b) H_3_O^+^; (c) BF_3_·Et_2_O, 30 °C.

The routes for the synthesis of 3-arylbenzofuranone derivatives are shown in Scheme 1 and the final products are listed in Table 1. The synthesis method of the 3-arylbenzofuranone compound was based on Lv et al. and optimised slightly^21^. Substituted mandelic acid **3a–3d** were synthesised with substituted benzaldehyde **1a–1d**. The substituted mandelic acid and substituted phenol were used as starting materials to obtain the corresponding compounds **1**–**23** by esterification and intramolecular alkylation reaction. Details on the chemical and spectroscopic characterisations of compounds **1**–**23** were described in the Supporting Information.

**Table 1.** Compounds **1**–**23**.

Product	R_1_	R_2_	R_3_	R_4_	R_5_	R_6_	R_7_
**1**	H	H	OCH_3_	H	H	OH	H
**2**	H	H	OCH_3_	H	H	H	OH
**3**	H	H	OCH_3_	H	H	OCH_3_	H
**4**	H	H	OCH_3_	H	H	H	OCH_3_
**5**	H	H	OCH_3_	H	OH	OH	H
**6**	H	OCH_3_	OCH_3_	H	H	OH	H
**7**	H	H	OCH_3_	H	H	OCH_3_	OH
**8**	H	H	OCH_3_	H	OH	OCH_3_	H
**9**	H	OCH_3_	OCH_3_	H	H	OCH_3_	H
**10**	H	H	OCH_3_	H	H	OCH_3_	OCH_3_
**11**	H	OCH_3_	OCH_3_	OCH_3_	H	OH	H
**12**	H	OCH_3_	OCH_3_	H	OH	OCH_3_	H
**13**	H	OCH_3_	OCH_3_	H	H	OCH_3_	OH
**14**	H	OCH_3_	OCH_3_	OCH_3_	H	H	OH
**15**	H	OCH_3_	OCH_3_	OCH_3_	H	H	OCH_3_
**16**	H	OCH_3_	OCH_3_	OCH_3_	H	OCH_3_	H
**17**	OCH_3_	OCH_3_	OCH_3_	H	H	H	OCH_3_
**18**	OCH_3_	OCH_3_	OCH_3_	H	H	OCH_3_	H
**19**	H	OCH_3_	OCH_3_	H	H	OCH_3_	OCH_3_
**20**	H	OCH_3_	OCH_3_	OCH_3_	OH	OH	H
**21**	H	OCH_3_	OCH_3_	OCH_3_	H	OCH_3_	OH
**22**	H	OCH_3_	OCH_3_	OCH_3_	OH	OCH_3_	H
**23**	H	OCH_3_	OCH_3_	OCH_3_	H	OCH_3_	OCH_3_

Compared with the original method, the ratio of mandelic acid compounds to phenolic compounds was adjusted from 1:1 to 1:1.2 to ensure that the mandelic acid compounds fully reacted. The completion of the reaction is monitored by thin layer chromatography (TLC), not just the reaction time. Compounds with different substituents differ greatly in reaction time. Excessive reactions can affect compound yield and purity. Several polyhydroxy compounds were synthesised by microwave reactions. The previous reaction by heating in an oil bath required a reaction for 8 h, and now the reaction time is shortened to 15 min by 200 W microwave heating. The yield of the compound obtained by microwave heating is also slightly improved. Most 3-arylbenzofuranone compounds can be purified by methanol recrystallisation, which is efficient and fast.

### Biology

#### *In vitro* ChE inhibitory activity

AChE is responsible for the hydrolysis of ACh in the synaptic cleft, and AChE inhibitors may help increase ACh levels in damaged cholinergic neurons^22^. Some of the most common untoward effects of ChEs inhibition therapy are gastrointestinal complaints resulting from stimulation of peripheral autonomic cholinergic systems. The pre-clinical data indicate that more selective AChE inhibitors produce fewer peripheral cholinergic signs than non-selective ChE inhibitors^23^. Dual ChE inhibitors may increase the incidence of adverse reactions.

The ChE inhibitory activity of all compounds was evaluated by the method of Ellman^16^. Donepezil was used as a reference compound in this assay. As shown in Table 2, all compounds presented AChE inhibitory activity. Notably, compound **20** (IC_50_ = 0.089 ± 0.005 μM) had relatively strong activity, which displayed little weaker capacity than donepezil (IC_50_ = 0.059 ± 0.003 μM). Compounds **4**, **5**, **7**, **9**, **13**, **20** have IC_50_ values less than 1 μM. Compound **1** (IC_50_ = 50.58 ± 3.12 μM) and **11** (IC_50_ = 48.24 ± 0.34 μM) were respectively lacking one hydroxyl group at R_5_ than compound **5** (IC_50_ = 0.48 ± 0.05 μM) and **20** (IC_50_ = 0.089 ± 0.005 μM), so we speculated that the R_5_, R_6_-dihydroxy substituted compound is better than the R_6_ monosubstituted compound at AChE inhibitory activity. The activities of the compounds **7** (IC_50_ = 0.52 ± 0.07 μM), **13** (IC_50_ = 0.28 ± 0.01 μM) are respectively superior to those of the compounds **10** (IC_50_ = 28.65 ± 1.44 μM), **19** (IC_50_ = 36.50 ± 0.51 μM), so we presumed that the R_6_ methoxy-substituted and the R_7_ hydroxy-substituted compound is superior to that of the R_6_, R_7_-dimethoxy-substituted compound. Nearly half of the compounds have BuChE inhibitory activity. The IC_50_ value of compound **13** (IC_50_ = 9.91 ± 0.48 μM) is similar to positive drug donepezil (IC_50_ = 4.67 ± 0.16 μM). Compounds **2**, **7**, and **13** have IC_50_ values less than 50 μM. As shown in Table 2, most of the compounds have selective AChE inhibitory activity, which may have a lower incidence of adverse reactions than the double ChE inhibitor.

**Table 2.** Biological evaluation *in vitro*.

Product	IC_50_ value (μM)			
	AChE inhibitory activity	BuChE inhibitory activity	MAO-B inhibitory activity	Antioxidant activity
**1**	50.58 ± 3.12	>200	61.26 ± 0.79	13.28 ± 0.27
**2**	4.15 ± 1.02	24.92 ± 0.96	14.67 ± 0.78	>100
**3**	80.48 ± 3.41	>200	298.43 ± 5.4	11.78 ± 0.18
**4**	0.74 ± 0.06	>200	383.45 ± 5.08	33.07 ± 1.45
**5**	0.48 ± 0.05	>200	22.48 ± 0.22	5.51 ± 0.20
**6**	24.38 ± 1.21	>200	48.59 ± 2.02	/
**7**	0.52 ± 0.07	32.92 ± 0.76	91.13 ± 0.70	2.62 ± 0.21
**8**	9.24 ± 0.62	>200	40.62 ± 1.92	5.96 ± 0.14
**9**	0.35 ± 0.03	>200	354.19 ± 5.05	17.17 ± 0.58
**10**	28.65 ± 1.44	>200	493.39 ± 7.10	13.89 ± 0.11
**11**	48.24 ± 0.34	>200	38.61 ± 0.42	11.22 ± 0.24
**12**	5.25 ± 0.44	180.91 ± 1.48	22.85 ± 0.73	1.60 ± 0.10
**13**	0.28 ± 0.01	9.91 ± 0.48	11.24 ± 0.72	5.33 ± 0.08
**14**	3.37 ± 0.17	128.33 ± 4.65	69.65 ± 1.26	5.11 ± 0.13
**15**	2.54 ± 0.19	>200	215.22 ± 2.27	32.58 ± 0.42
**16**	4.07 ± 0.19	>200	>500	18.01 ± 0.48
**17**	42.26 ± 4.01	>200	82.68 ± 1.31	29.17 ± 0.31
**18**	123.88 ± 2.17	>200	180.34 ± 1.32	64.48 ± 1.29
**19**	36.50 ± 0.51	>200	145.56 ± 1.83	12.56 ± 0.14
**20**	0.089 ± 0.01	>200	149.21 ± 3.39	42.56 ± 2.58
**21**	2.38 ± 0.20	145.89 ± 3.05	>500	5.35 ± 0.33
**22**	2.03 ± 0.09	>200	30.27 ± 0.65	6.44 ± 0.12
**23**	25.96 ± 1.26	>200	43.89 ± 1.26	11.94 ± 0.16
Donepezil	0.059 ± 0.003	4.67 ± 0.16		
Rasagiline			0.104 ± 0.002	
Ascorbic acid				7.69 ± 0.10

Each value represents the mean ± SD (*n* = 3).

#### *In vitro* MAO inhibitory activity

MAO plays a major role in brain development and function, and its inhibitors are used clinically as antidepressants and anti-Parkinson’s drugs. MAO-B activity is increased in the brains of Alzheimer’s patients^24^. All the synthesised compounds were evaluated for their MAO inhibitory activity in the way of Holt by references^17^. Among them, compound **13** (IC_50_ = 11.24 ± 0.72 μM) had the best inhibitory activity of MAO-B but was still weaker than the positive drug rasagiline (IC_50_ = 0.104 ± 0.002 μM). Compounds **8**, **12**, **22** all have a hydroxyl group at R_5_ and a methoxy group at the R_6_ position, all exhibiting good activity. All of the tested compounds showed good activity in the substitution of R_5_ with a hydroxyl group, so we speculated that the hydroxyl substitution at the R_5_ position may contribute to an increase in the MAO-B inhibitory activity of the compound.

#### *In vitro* antioxidant activity

Alzheimer’s patients exhibit extensive oxidative stress throughout the body. Oxidative stress is an early and prominent symptom of AD, which plays an important role in AD^20^. All the synthesised compounds were evaluated for their antioxidant activities in the way of scavenging DPPH^25^. The 3-arylbenzofuranone compounds exhibit good antioxidant capacity. The antioxidant activity of the compounds **5**, **7**, **8**, **12**, **13**, **14**, **21**, **22** was superior to the positive drug vitamin C (IC_50_ = 7.69 ± 0.10 μM). Compounds **8**, **12**, **22** are substituted at the R_5_ position with a hydroxy group and at the R_6_ position with a methoxy group. Compounds **7**, **13**, and **21** are substituted at the R_6_ with a methoxy group and at the R_7_ position with a hydroxy group. Therefore, we speculated that the hydroxymethoxy ortho-substitution facilitates the improvement of the antioxidant capacity of the compound.

#### Kinetic study of ChEs inhibition

**Figure 1.**
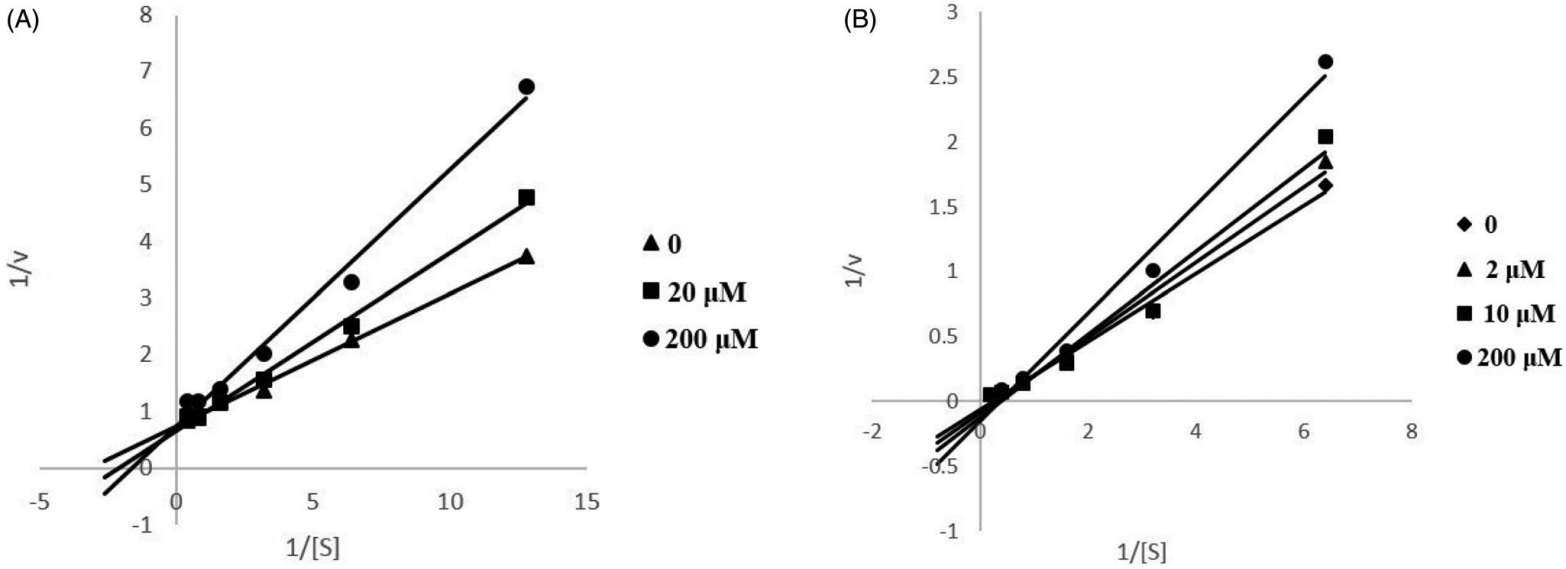
Kinetic study of the mechanism of ChEs inhibition by compound **13**. Overlaid Lineweaver–Burk reciprocal plots of ChEs initial velocity at increasing substrate concentration in the absence of inhibitor and in the presence of **13** are shown. A is a double reciprocal plot of compound **13** inhibition of AChE. B is a double reciprocal plot of compound **13** inhibition of BuChE.

In order to gain insight into the mechanism of action of these derivatives on ChEs, compound **13**, showing good inhibitory activity against ChEs, was selected for kinetic measurements. The graphical analysis of the steady-state inhibition data of **13** against ChEs is shown in Figure 1. According to the figure, it can be judged that the inhibition mode of compound **13** against ChEs is reversible inhibition.

#### Molecular modelling

**Figure 2.**
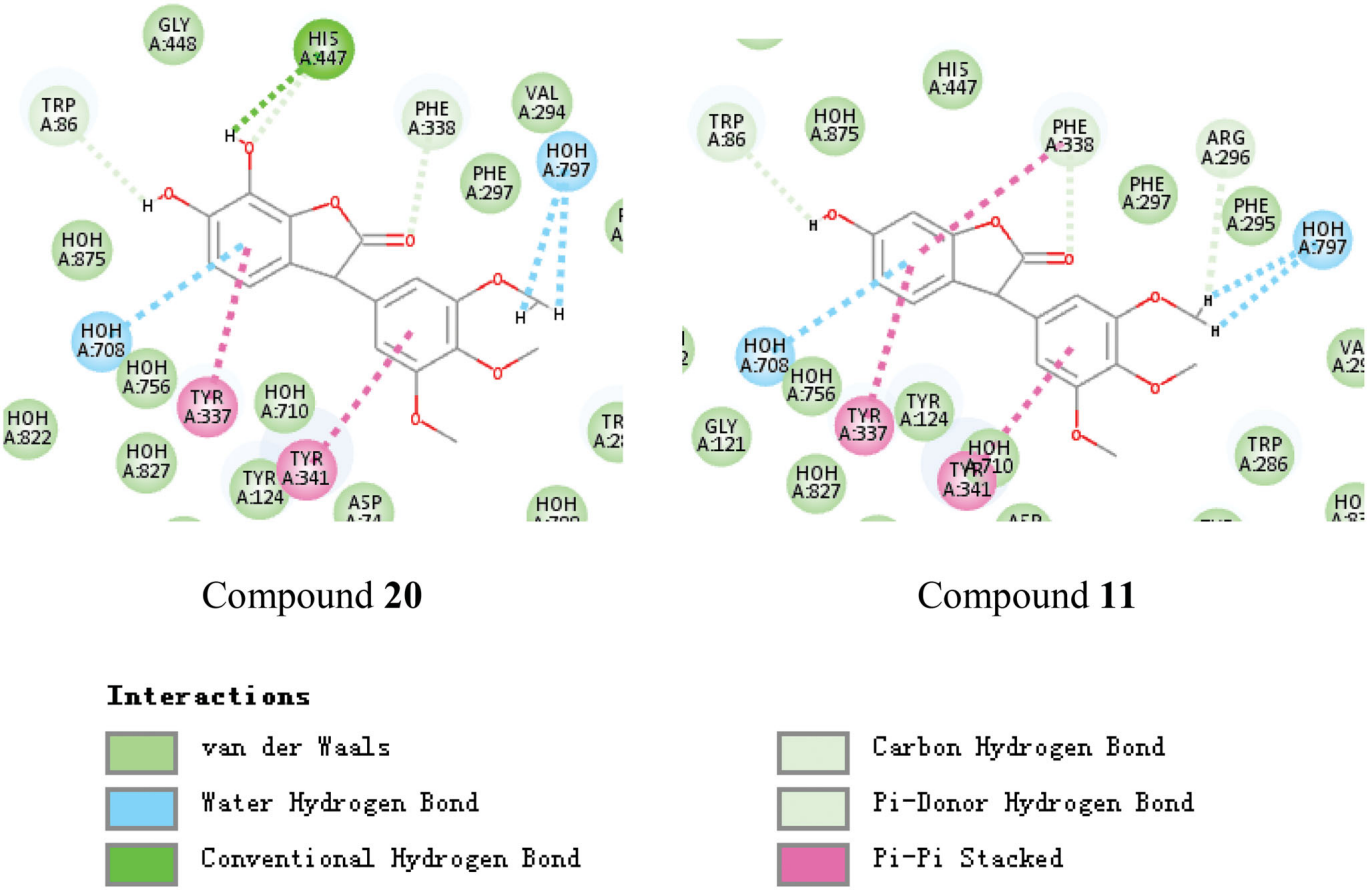
Schematic presentations of the putative AChE binding modes with compound **20** and compound **11**.

**Figure 3.**
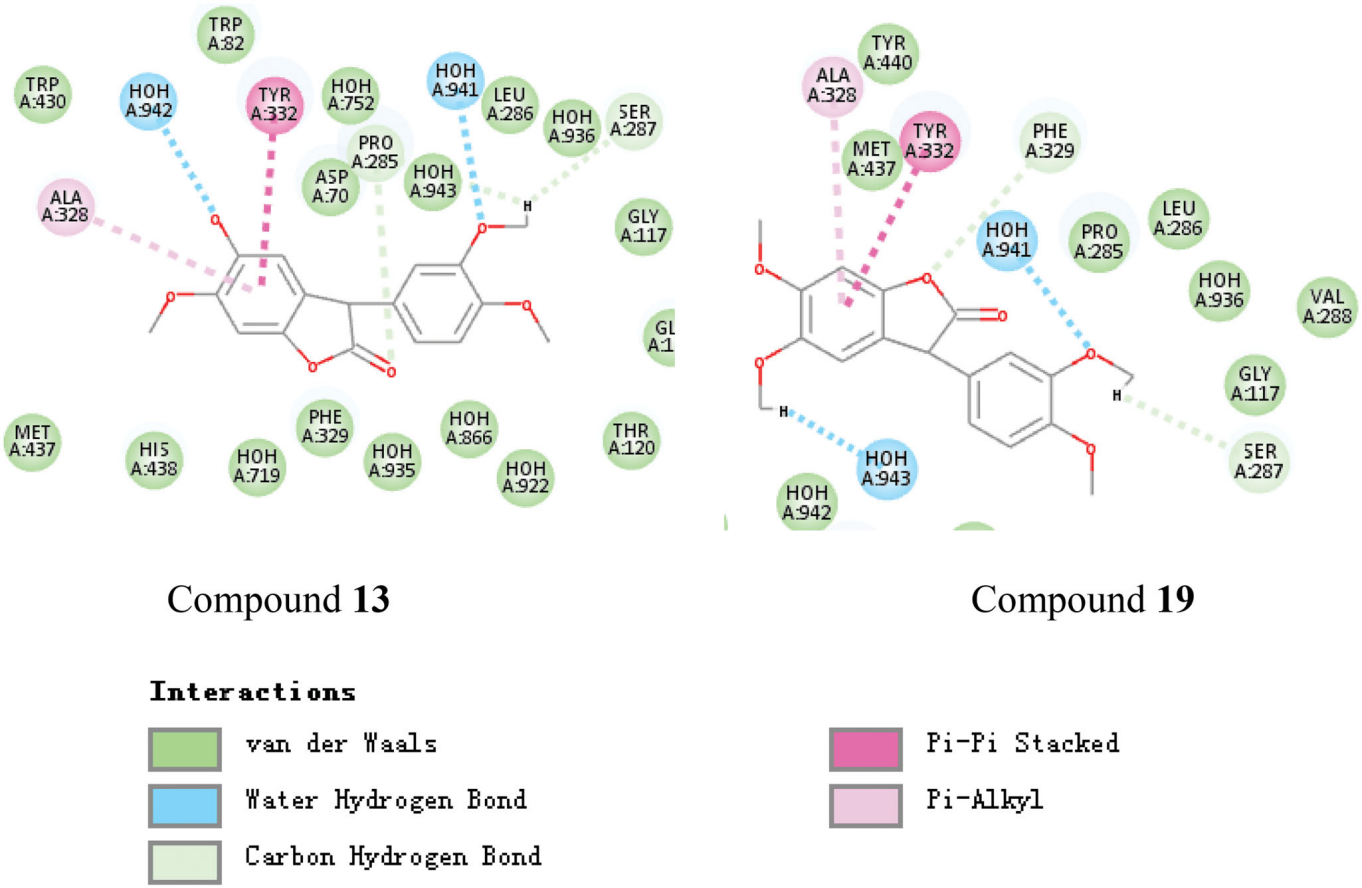
Schematic presentations of the putative BuChE binding modes with compound **13** and compound **19**.

**Figure 4.**
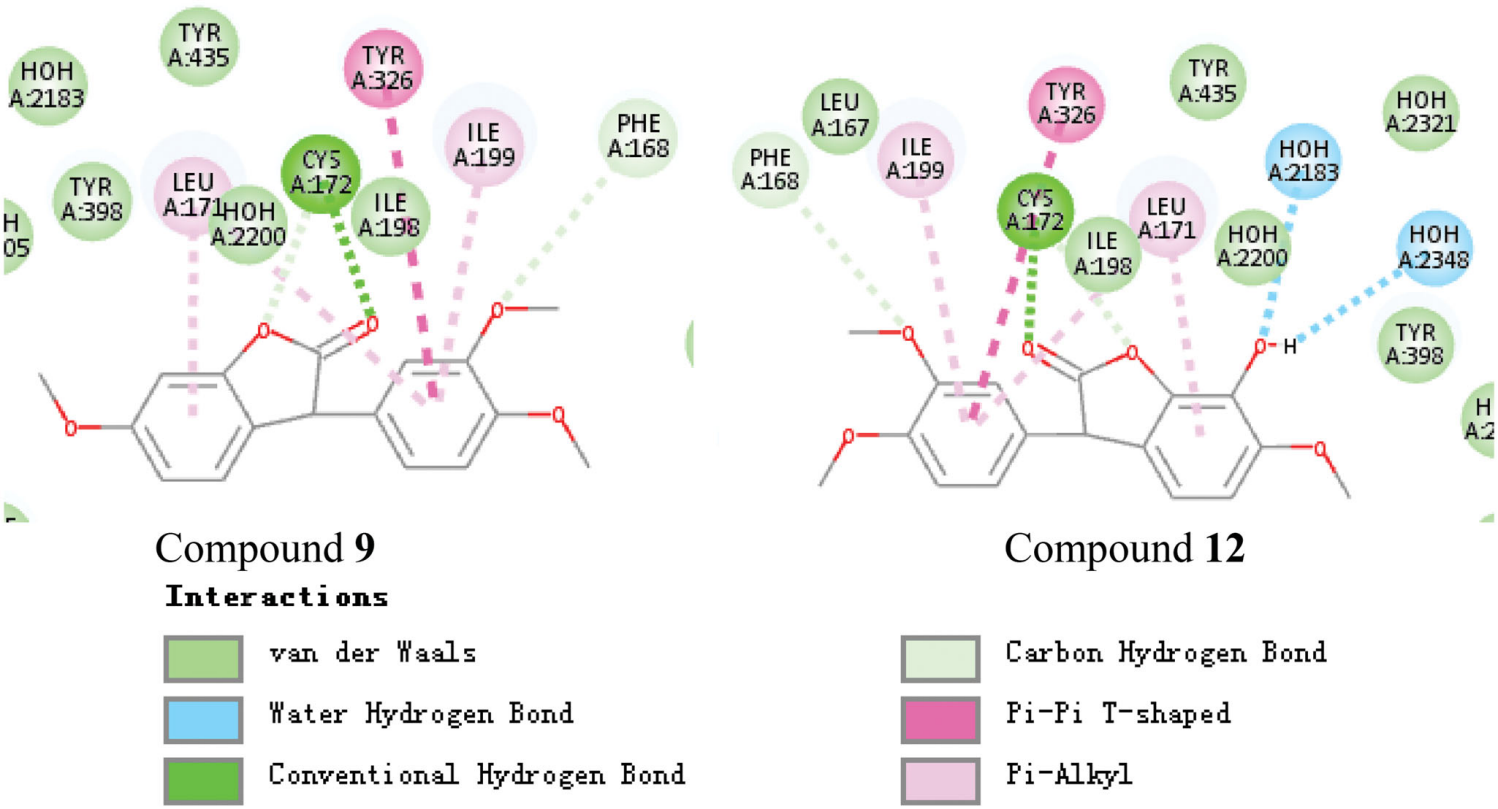
Schematic presentations of the putative MAO-B binding modes with compound **9** and compound **12**.

To clarify the mechanism of the compound’s inhibitory activity on the enzyme, the binding model of the active and inactive compounds to the active site was compared. The active site binding patterns of compounds **20**, **11**, and 4A79 are shown in Figure 2, respectively. It can be seen from Figure 2 that the main difference between the two compounds and the 4A79 binding model is the interaction between compound **20** and His447. This supports the R_5_, R_6_-dihydroxy substituted compound is better than the R_6_ monosubstituted compound at AChE inhibitory activity. The active site binding patterns of compounds **13**, **19**, and 6EP4 are shown in Figure 3, respectively. The effect of substituents on BuChE inhibition cannot be summarised by IC_50_ value and molecular docking results. The active site binding patterns of compounds **9**, **12**, and 4A79 are shown in Figure 4, respectively. It can be seen from Figure 4 that the main difference between the two compounds and the 6O4W binding model is the interaction between the hydroxyl group at the R_5_ position of compound **12** and two molecules of water. This supports the hydroxyl substitution at the R_5_ position may contribute to an increase in the MAO-B inhibitory activity of the compound.

#### TOPKAT prediction

Toxicity Prediction by Komputer assisted technology (TOPKAT) protocol in DS was used to predict the potential toxicity of the compound and the acute oral toxicity effects in rats. According to the analysis of the results in Table 3, all compounds were non-mutagenic. Table 3 summarises the acute oral toxicity effects of rats, indicating that a low level of toxicity with values ranging from 0.488 to 3.63 g/kg, which are all greater than the positive drug.

**Table 3.** TOPKAT prediction results

Compound	Mutagenicity	TD_50_ value (mg/kg)		LD_50_ value(g/kg)
		Carcinogenicity (Mouse)	Carcinogenicity (Rat)	Acute oral toxicity (Rat)
**1**	0.495	184	7.9	0.719
**2**	0.535	184	7.9	0.998
**3**	0.631	89	1.49	0.784
**4**	0.586	89	2.08	1.95
**5**	0.569	83.6	7.11	0.842
**6**	0.406	252	11.8	2.62
**7**	0.583	209	5.81	0.488
**8**	0.571	127	5.81	0.732
**9**	0.522	100	2.09	2.89
**10**	0.528	100	2.09	1.62
**11**	0.211	268	6.87	1.76
**12**	0.522	268	8.55	1.83
**13**	0.533	268	8.55	1.75
**14**	0.274	268	9.24	1.37
**15**	0.323	106	1.64	1.76
**16**	0.33	106	2.08	1.73
**17**	0.443	106	2.08	3.63
**18**	0.474	106	1.49	1.73
**19**	0.516	128	2.19	2.75
**20**	0.293	198	6.11	2.59
**21**	0.336	158	3.89	1.07
**22**	0.292	282	8.46	1.95
**23**	0.275	75.4	0.994	1.67
Donepezil	0.22	99.8	1.56	0.392
Rasagiline	0.598	94.9	20.2	0.427

## Conclusion

In conclusion, a series of 3-arylbenzofuranone derivatives were designed, synthesised, and evaluated as multi-targeting anti-AD agents, which have inhibitory activity against ChEs and MAO-B and antioxidant activity. All the synthesised compounds were evaluated for their antioxidant activities in the way of DPPH free radical scavenging experiment. Most compounds demonstrated moderate to high activity. According to the results of *in vitro* ChEs inhibition assay, we selected compound **13** with better ChE inhibition to study the kinetics of ChE inhibition. According to the kinetic experiments, the type of action of compound **13** on ChE inhibition is reversible inhibition, indicating that our design strategy is reasonable. Previously, our research group also carried out anti-AD research on 3-arylcoumarin compounds, and most of them have double ChE inhibitory activity^26^. Compared with 3-arylcoumarin compounds, 3-arylbenzofuranone compounds have better AChE and MAO-B inhibitory activity than 3-arylcoumarin compounds and have selective AChE inhibitory activity. Selective AChE compounds have fewer adverse reactions than double ChE inhibition, so screening for compounds with better selective AChE inhibitory activity also has a certain significance. Multi-target anti-AD compounds can modulate multiple signalling pathways or targets associated with AD, potentially producing significant clinical effects. All in all, the multifunctional effects of these 3-arylbenzofuranone derivatives qualify them as potential anti-AD agents, and they are more promising than 3-arylcoumarin compounds in anti-AD.

## Supplementary Material

Supplemental Material
