# Supplementary material for: Synthesis and biological evaluation of 3-arylbenzofuranone derivatives as potential anti-Alzheimer’s disease agents
Source: J Enzyme Inhib Med Chem. 2020 Mar 18;35(1):805–14. doi: 10.1080/14756366.2020.1740694 (PMC7155212; doi:10.1080/14756366.2020.1740694)

Synthesis and biological evaluation of 3-arylbenzofuranone derivatives as potential anti-Alzheimer's disease agents

Jie Yang, [†abcd] Yinling Yun, [†abcd] Yuhang Miao, [abcd] Jie Sun [*cde] and Xiaojing Wang [*cde]

[a] School of Medicine and Life Sciences, University of Jinan-Shandong Academy of Medical Sciences, Jinan 250200, Shandong, China
[b] Institute of MateriaMedica, Shandong Academy of Medical Sciences, Jinan 250062, Shandong, China
[c] Key Laboratory for Biotech-Drugs Ministry of Health, Jinan 250062, Shandong, China
[d] Key Laboratory for Rare & Uncommon Diseases of Shandong Province, Jinan 250062, Shandong, China
[e] Institute of Materia Medica, Shandong First Medical University ＆ Shandong Academy of Medical Sciences
* Correspondence: sunjie310@126.com (J.S.); xiaojing6@gmail.com (X.J.W.).
† These authors contributed equally to this work.

^{1}H NMR (600 MHz, DMSO-d_6) and ^{13}C NMR (151 MHz, DMSO-d_6) of compounds **1-23**.

compound **1** ^{1}HNMR

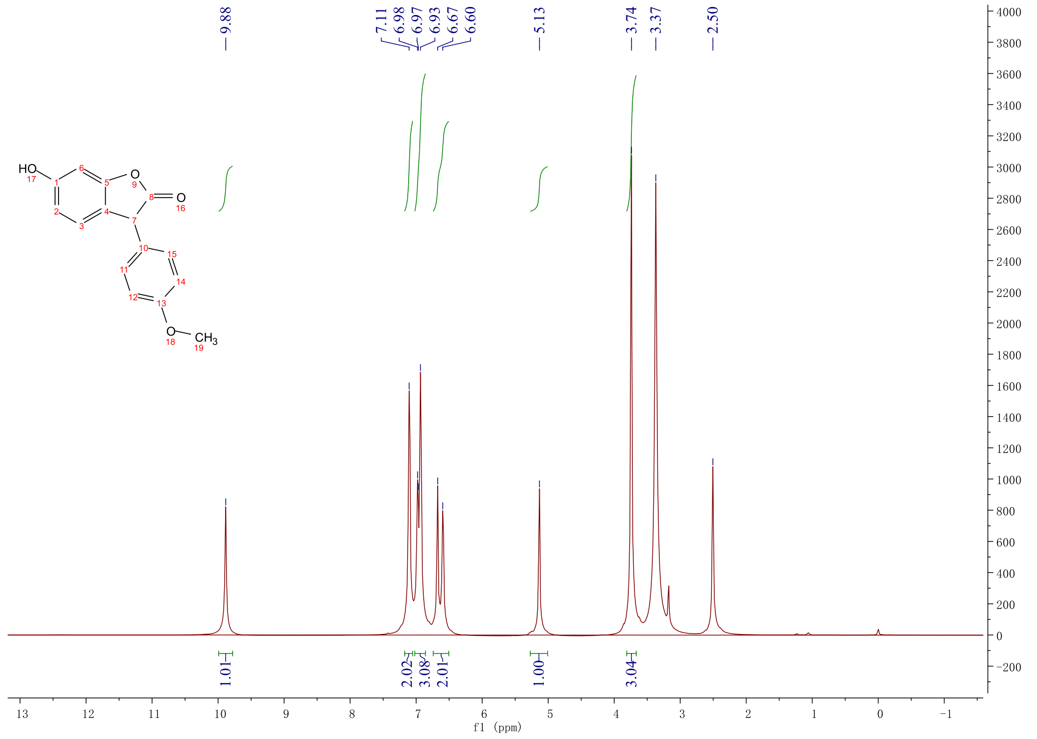

compound **1** ^{13}CNMR

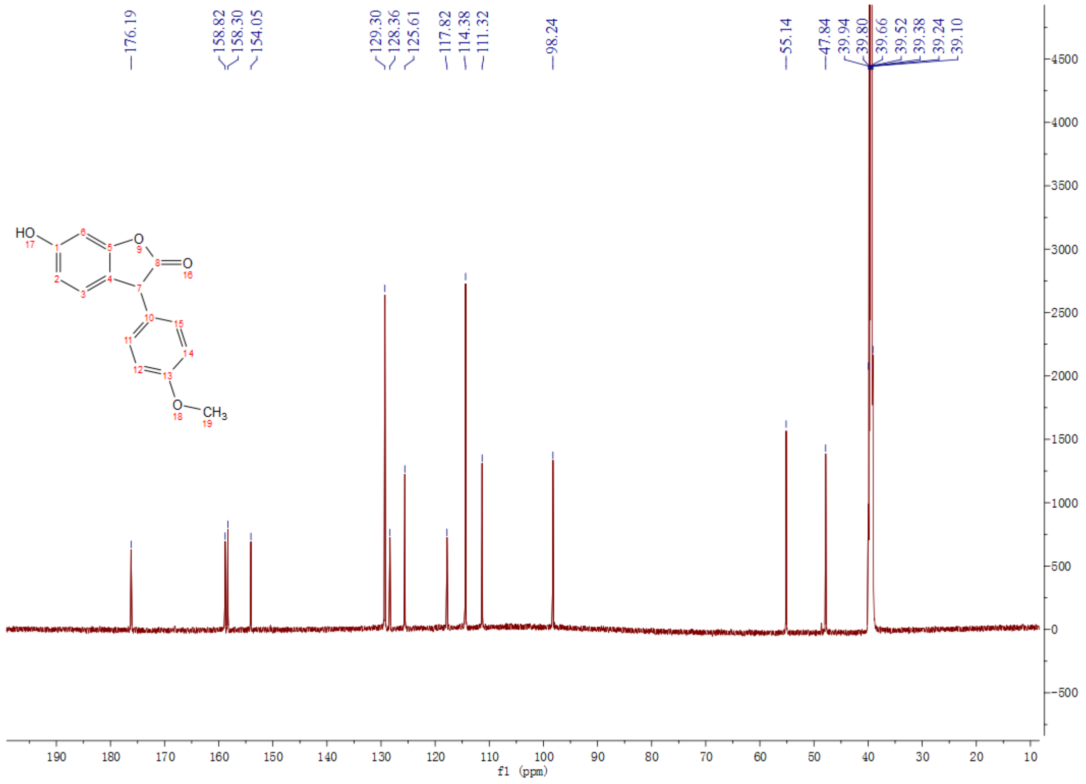

compound **2** ^{1}HNMR

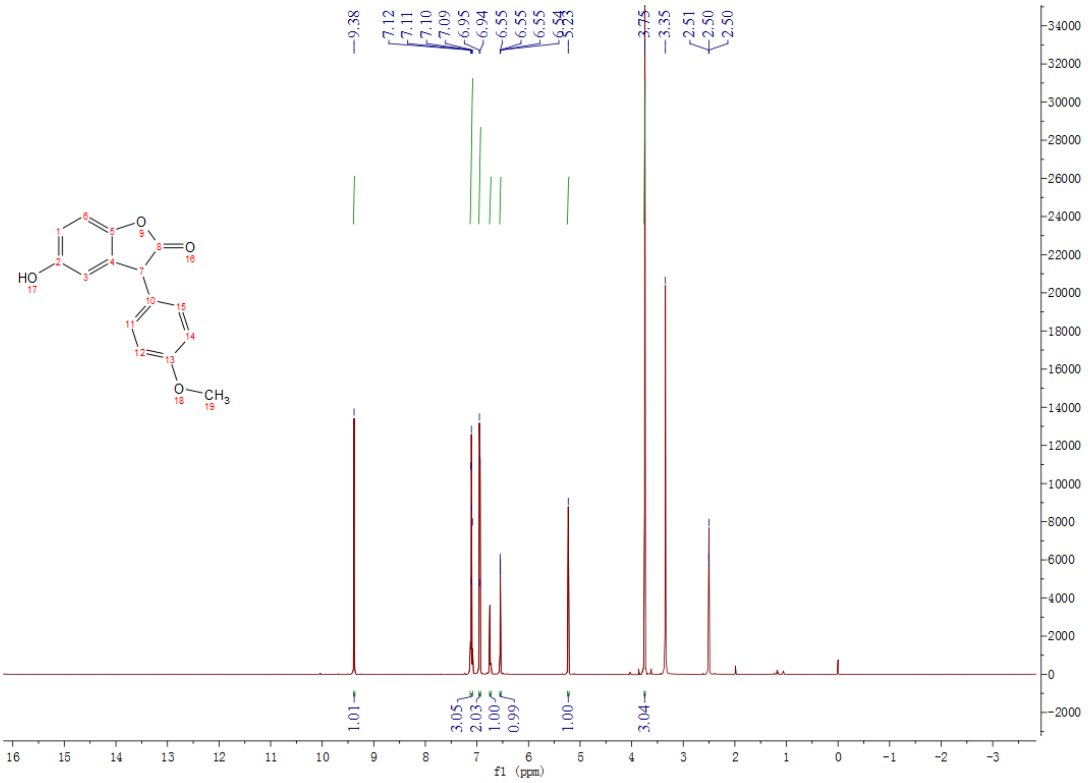

compound **2** ^{13}CNMR

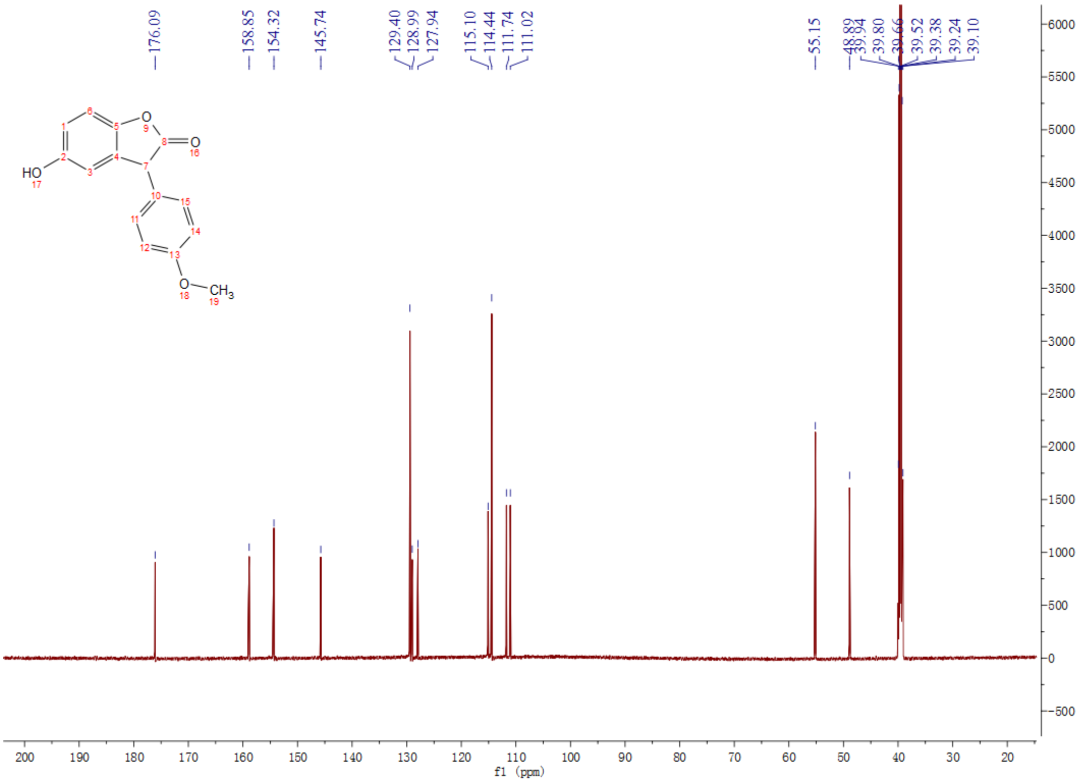

compound **3** ^{1}HNMR

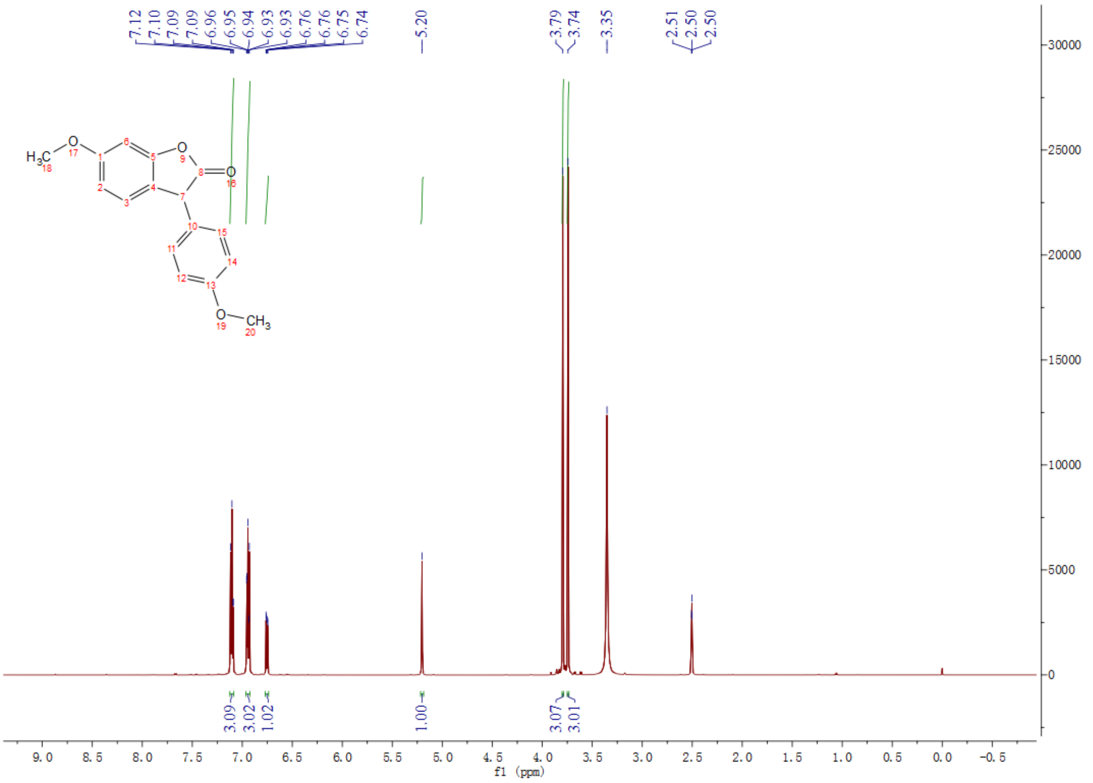

compound **3** ^{13}CNMR

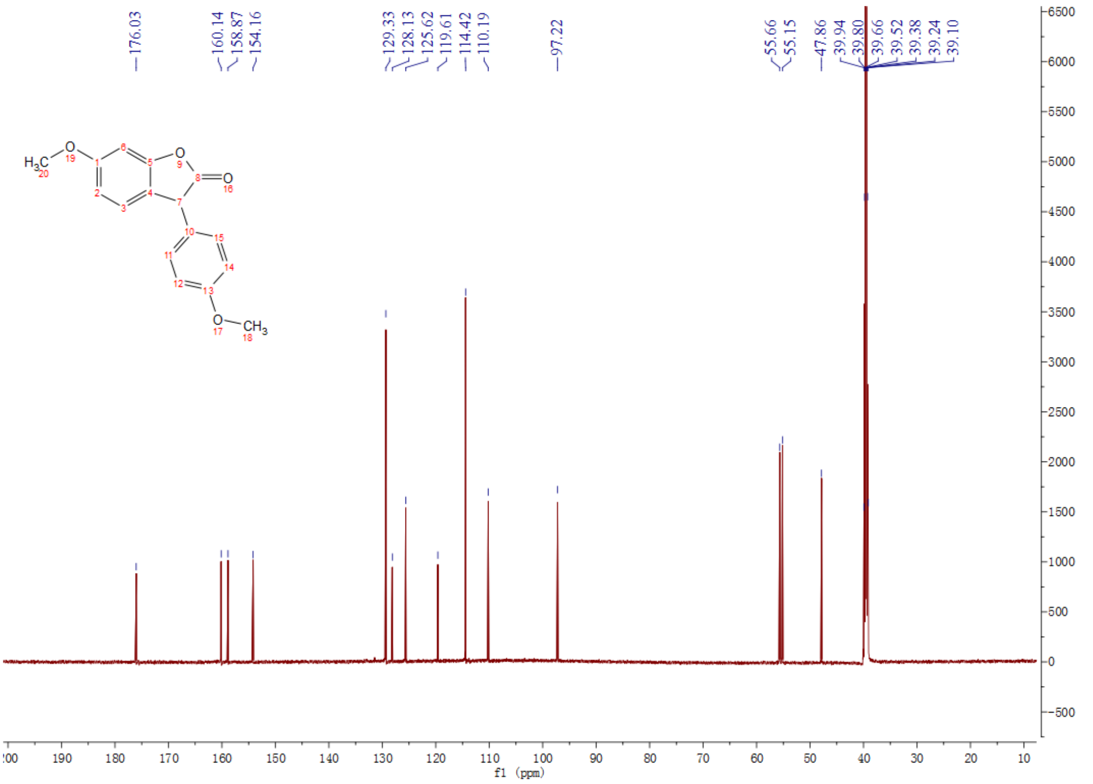

compound **4** ^{1}HNMR

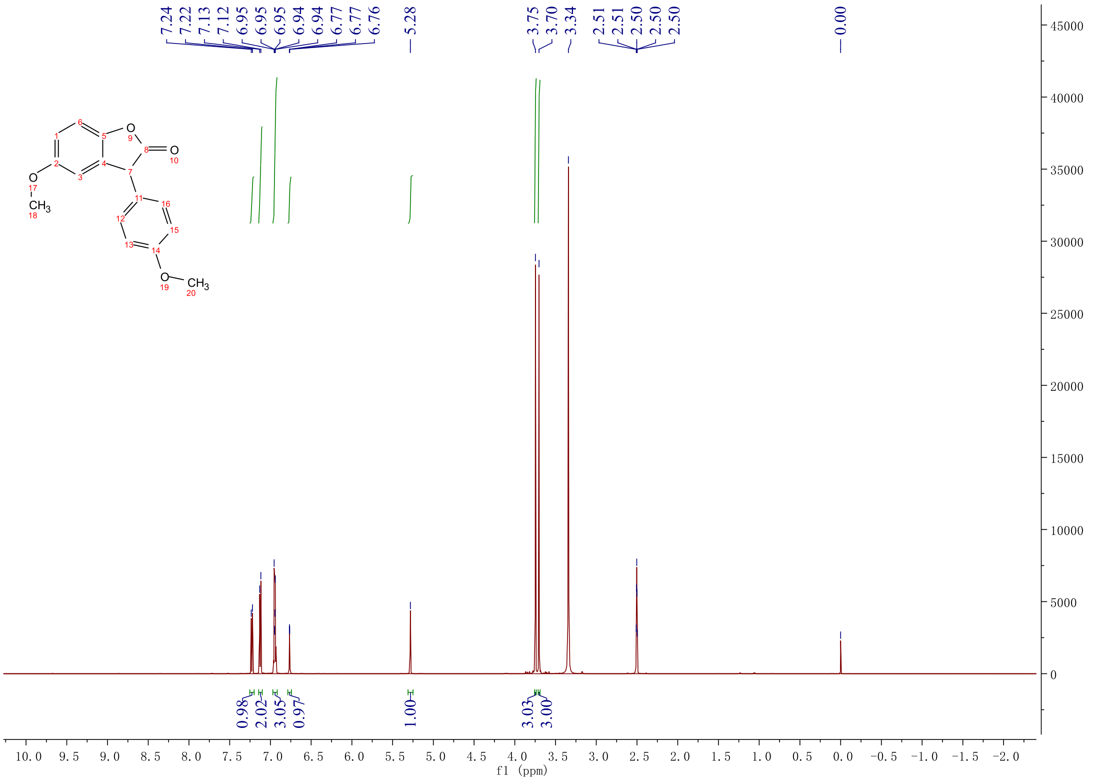

compound **4** ^{13}CNMR

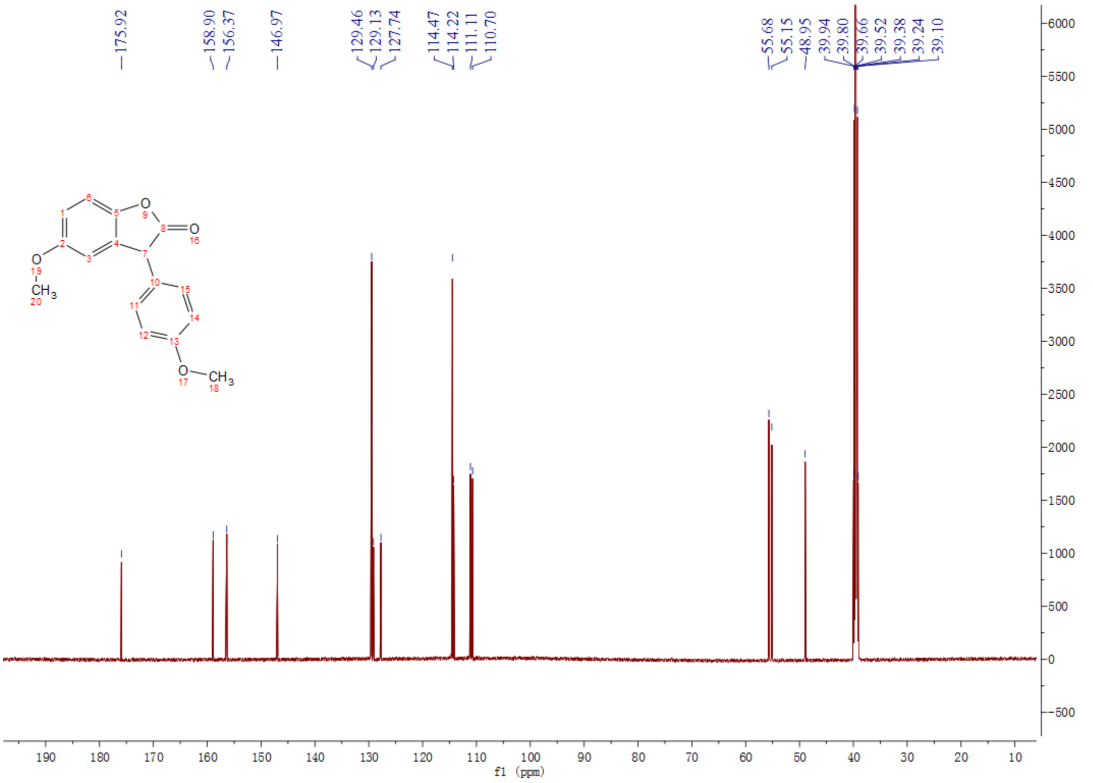

compound **5** ^{1}HNMR

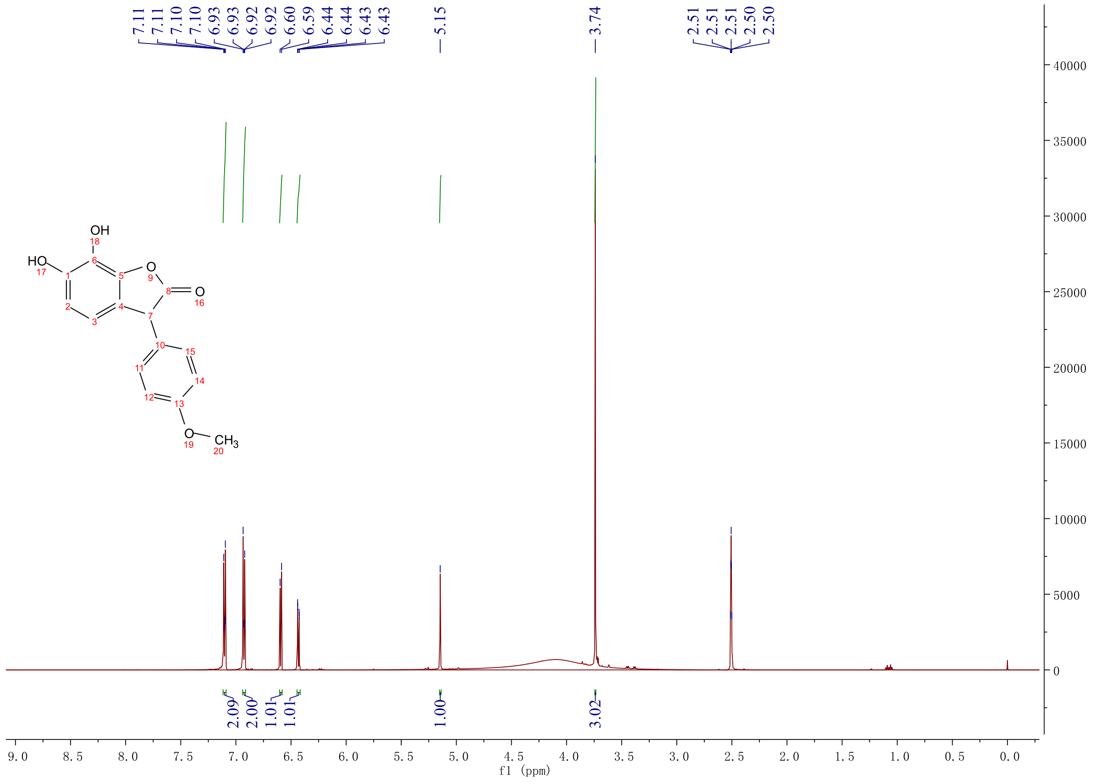

compound **5** ^{13}CNMR

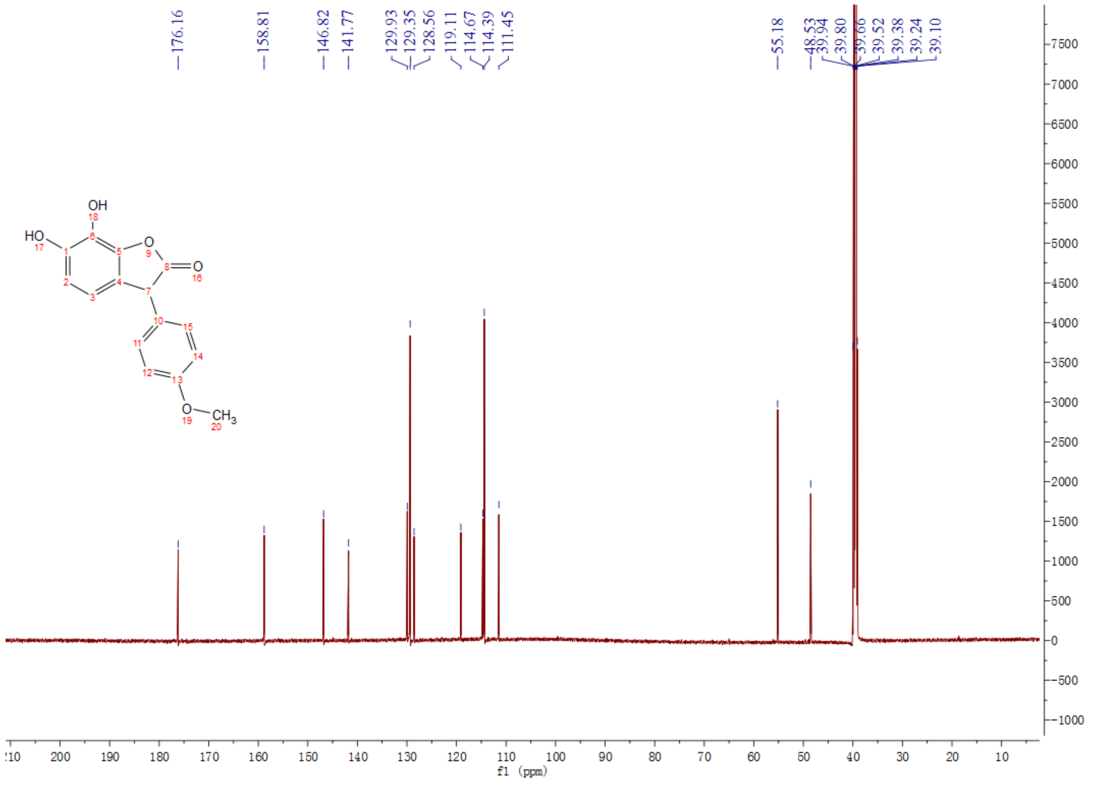

compound **6** ^{1}HNMR

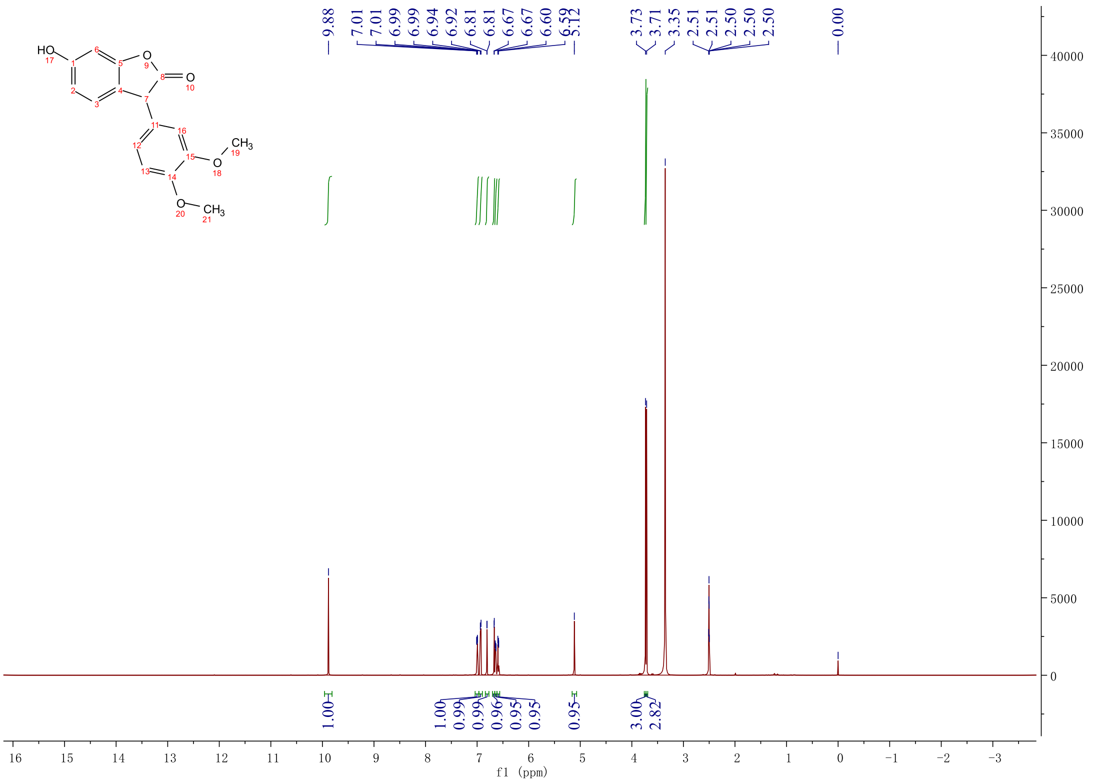

compound **6** ^{13}CNMR

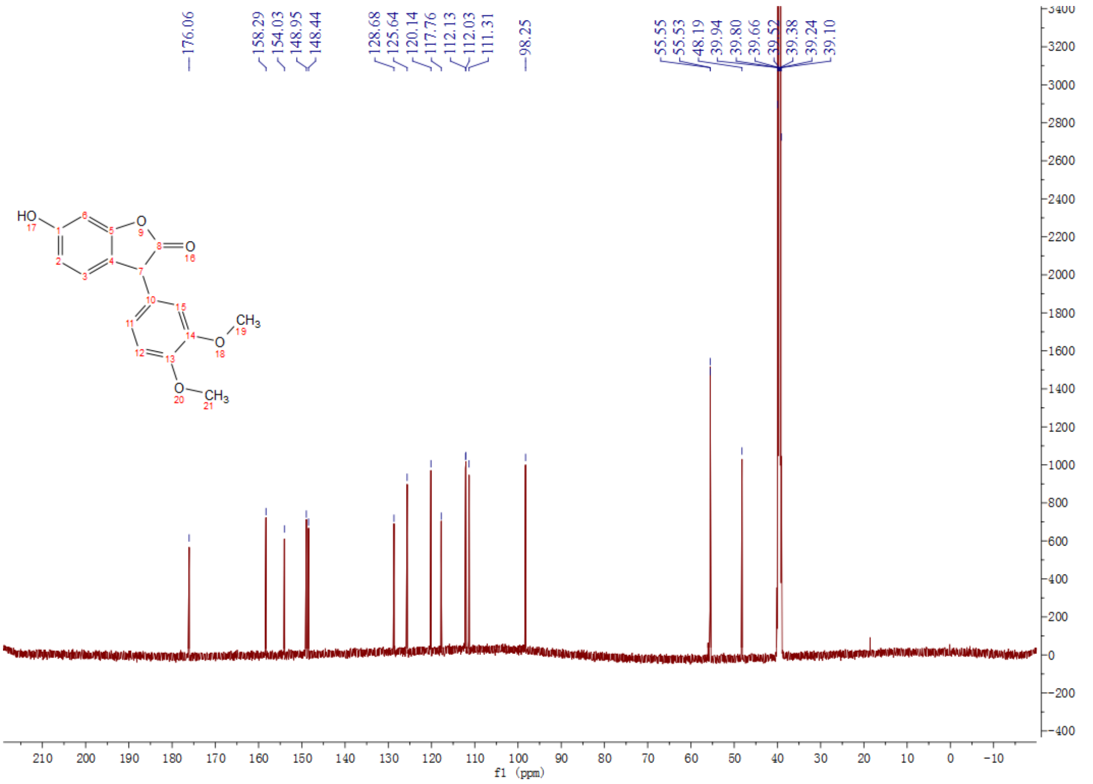

compound **7** ^{1}HNMR

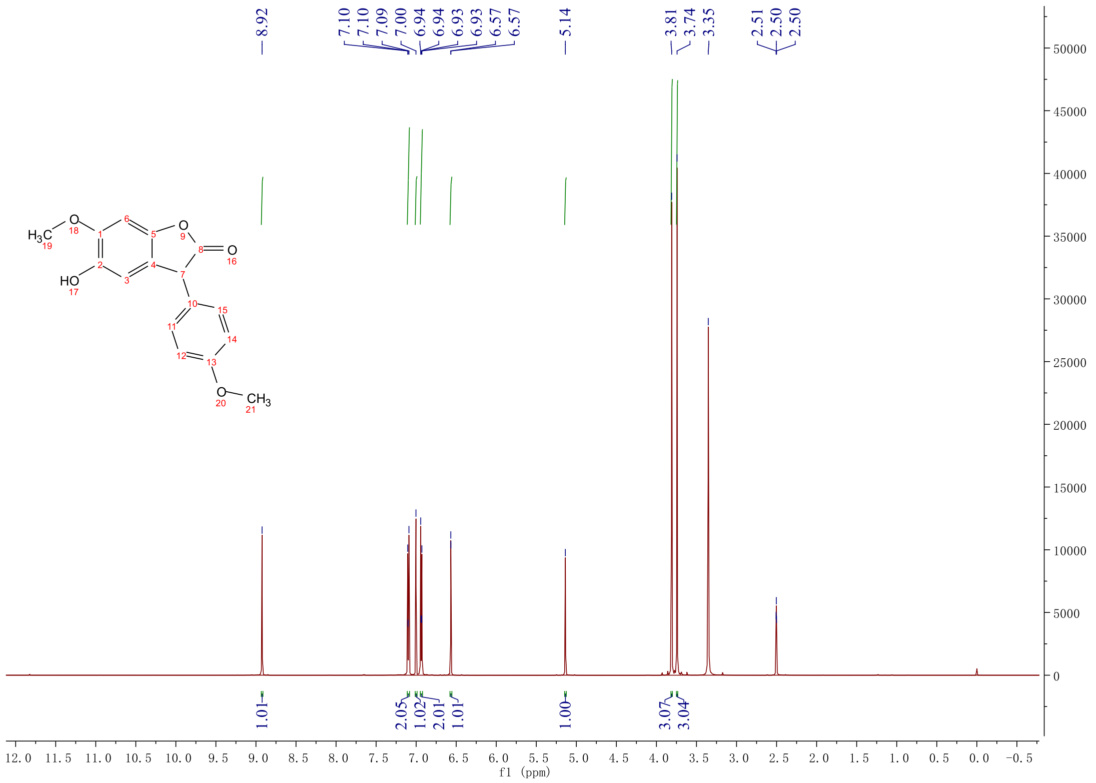

compound **7** ^{13}CNMR

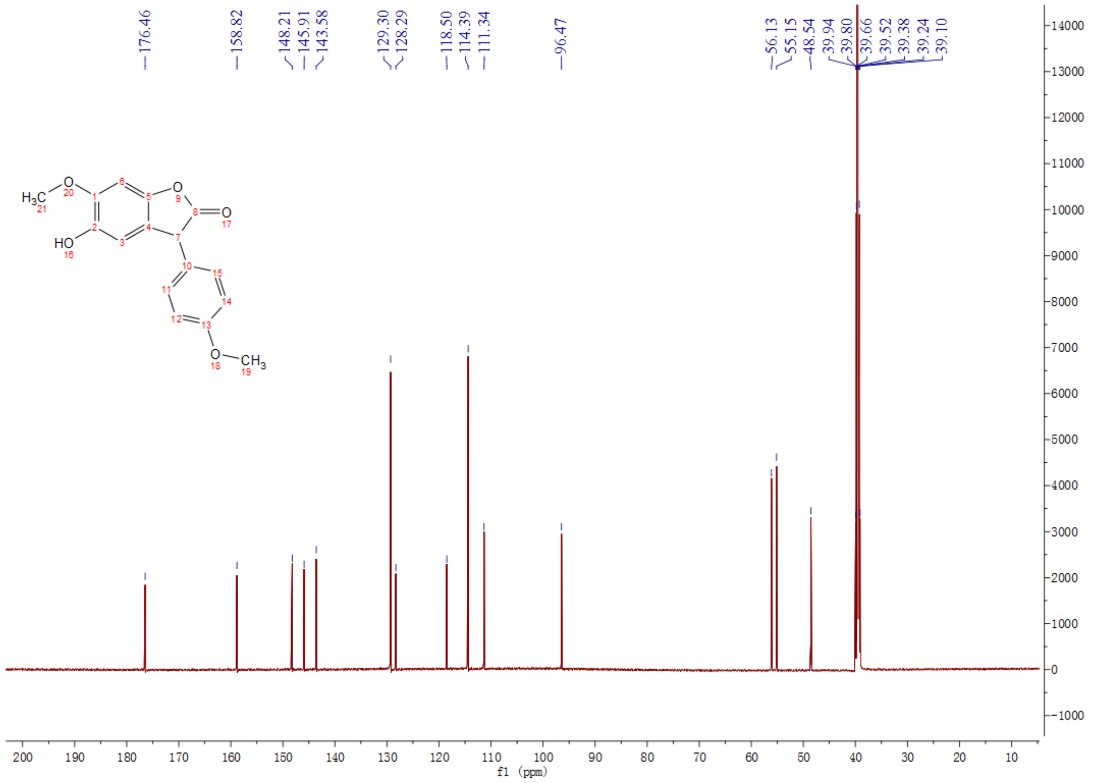

compound **8** ^{1}HNMR

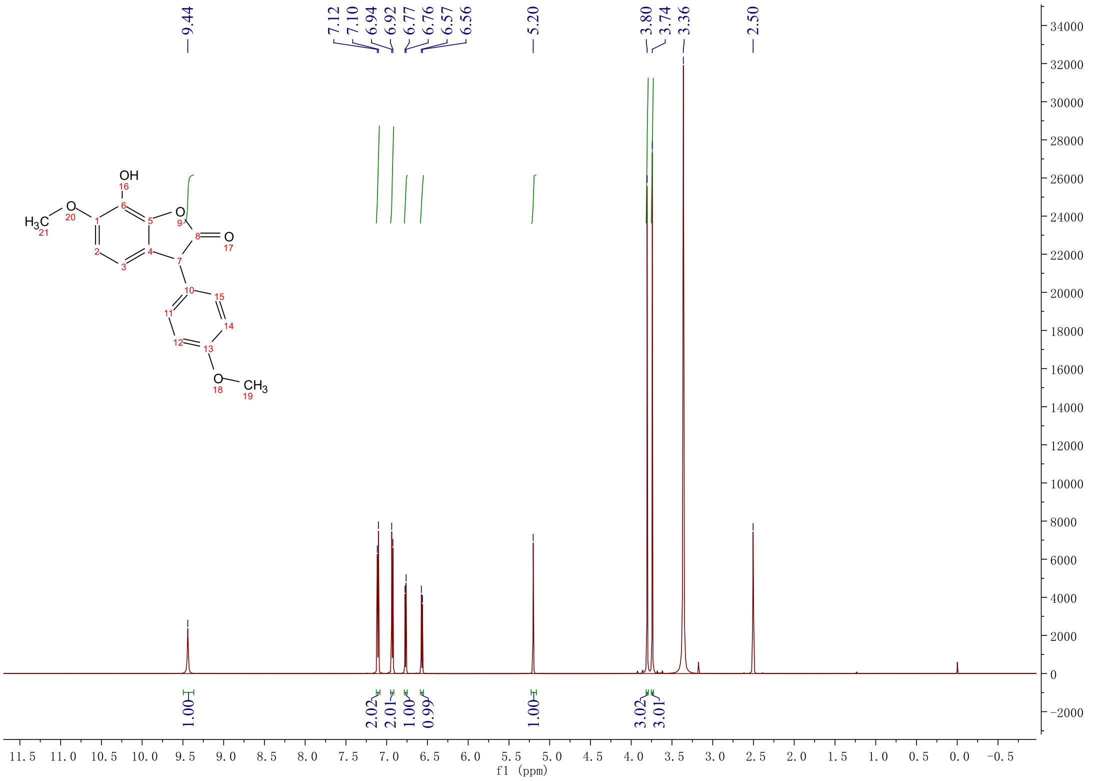

compound **8** ^{13}CNMR

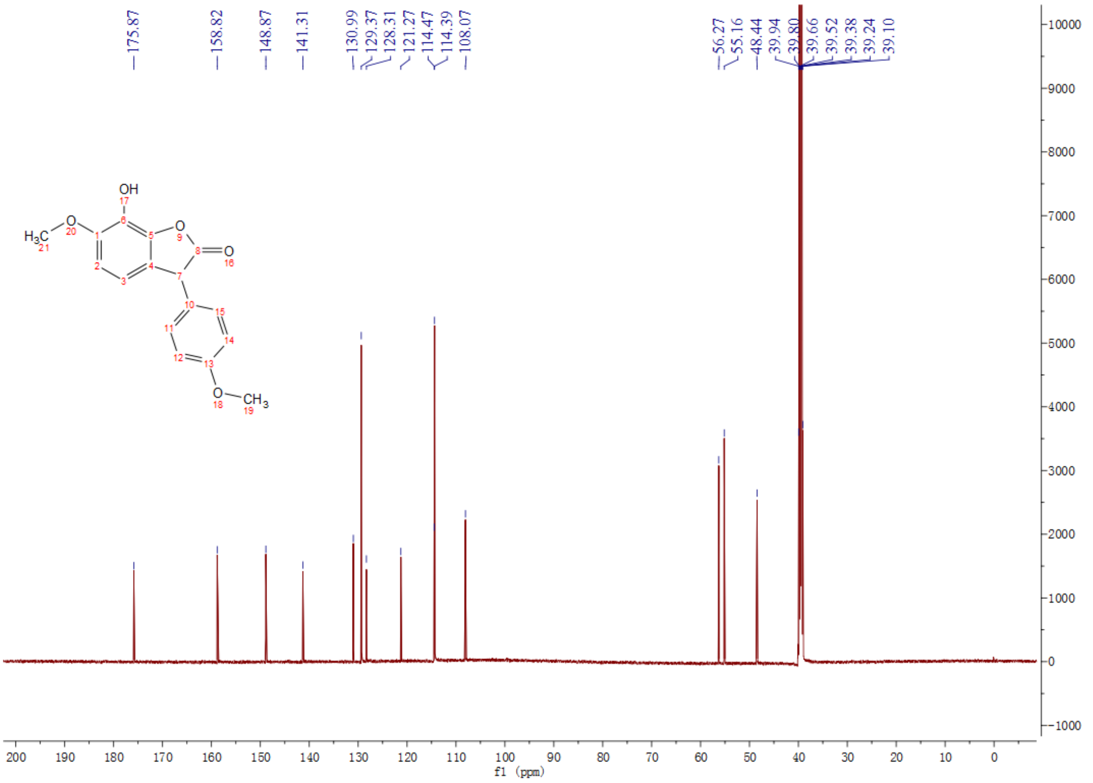

compound **9** ^{1}HNMR

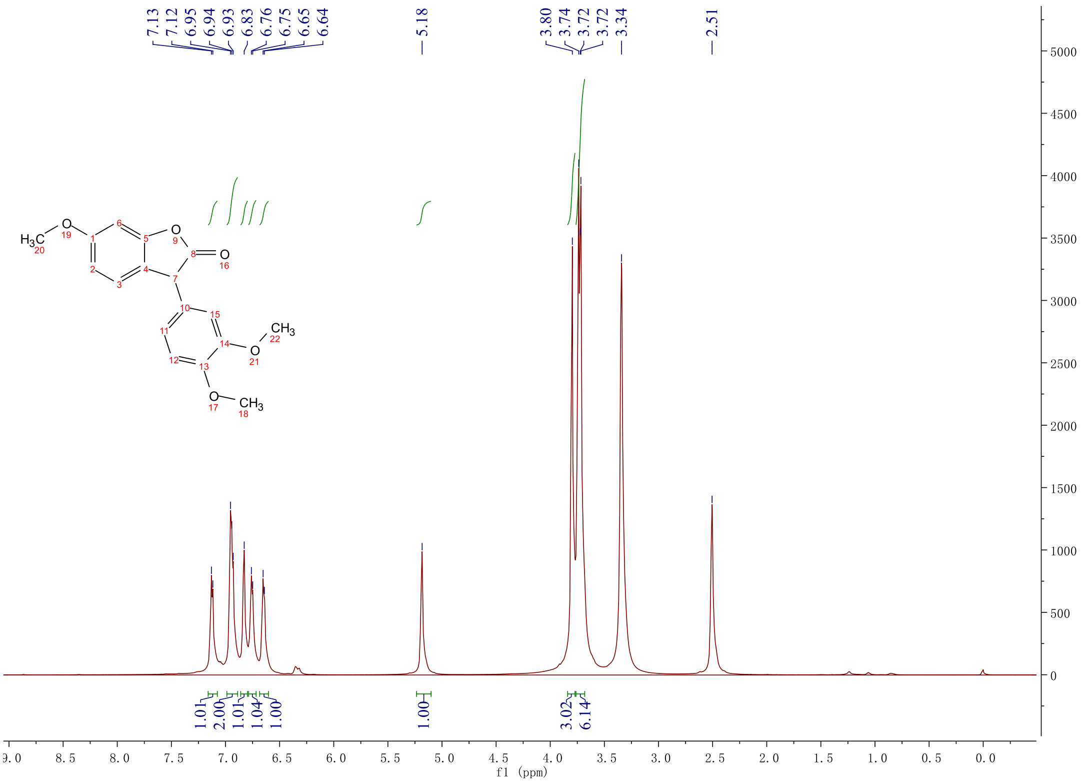

compound **9** ^{13}CNMR

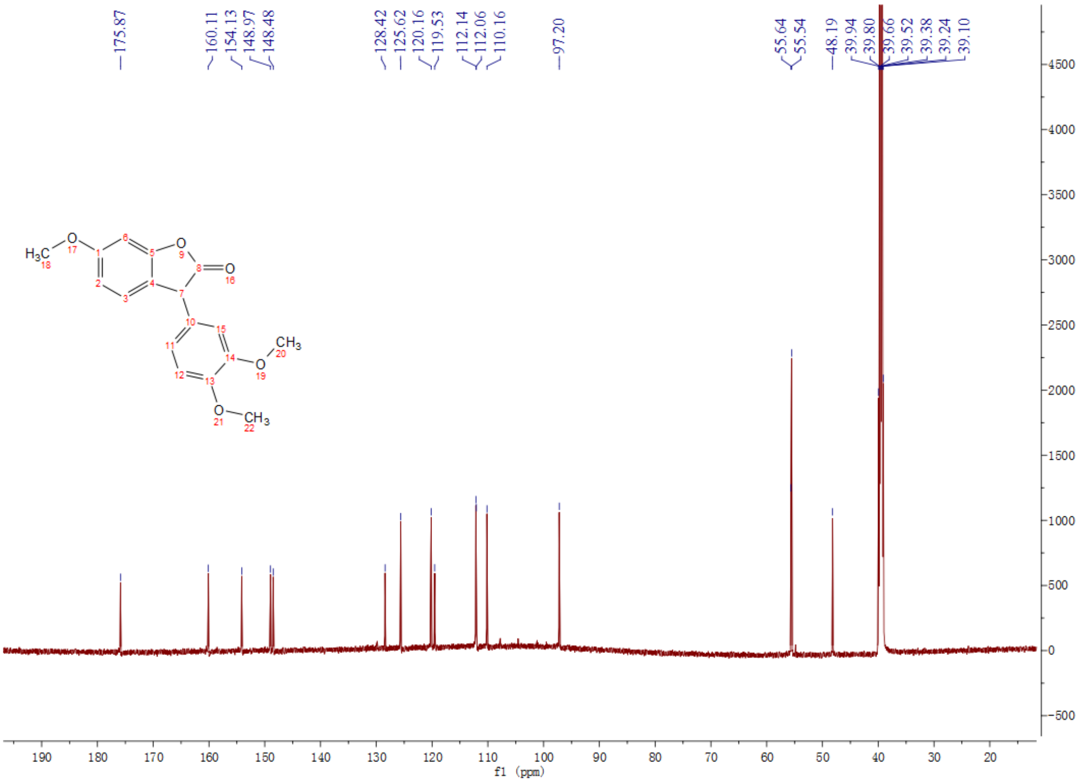

compound **10** ^{1}HNMR

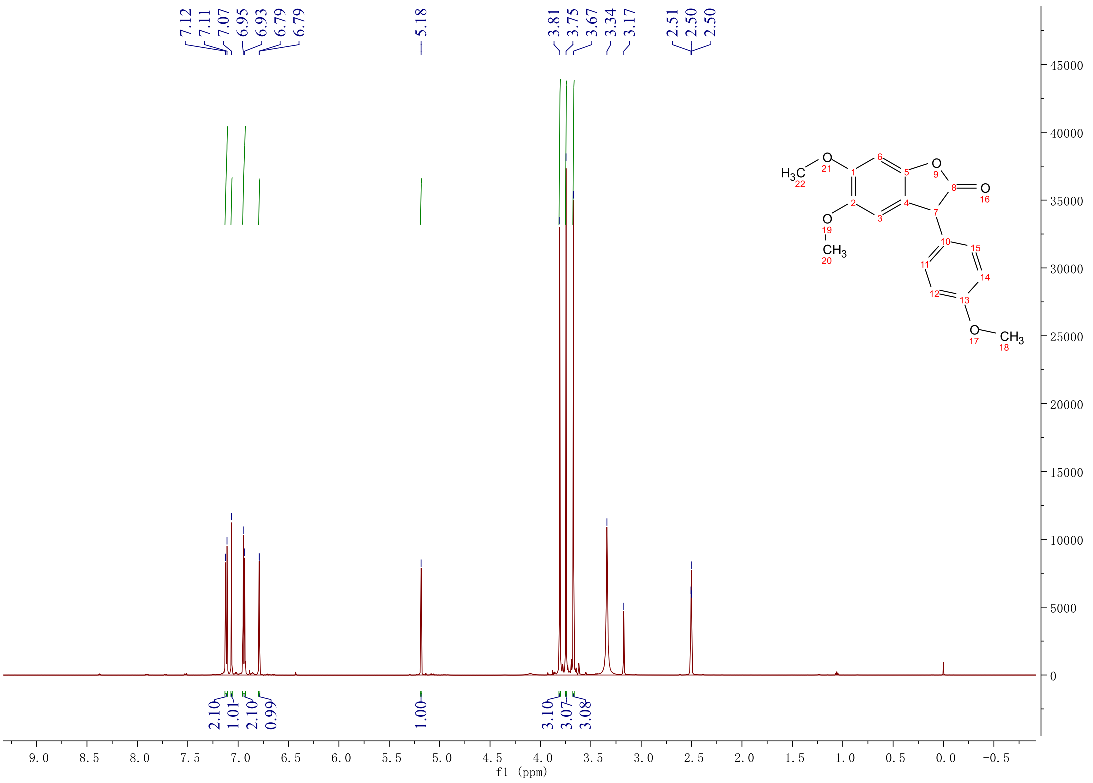

compound **10** ^{13}CNMR

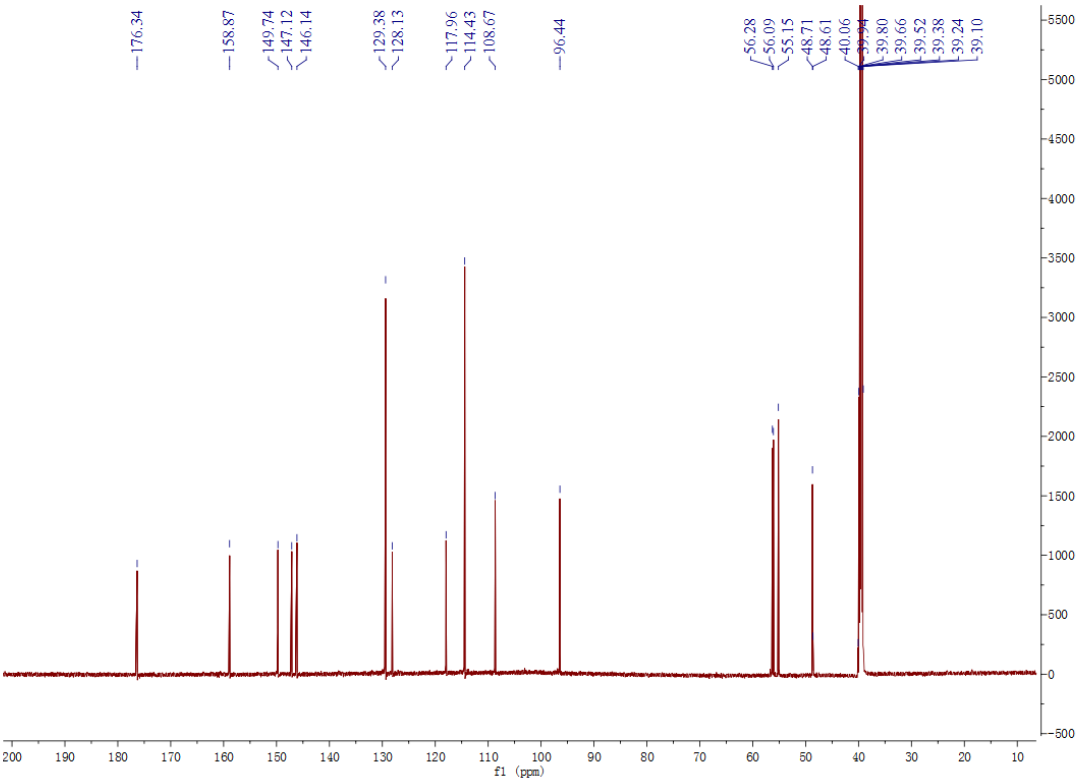

compound **11** ^{1}HNMR

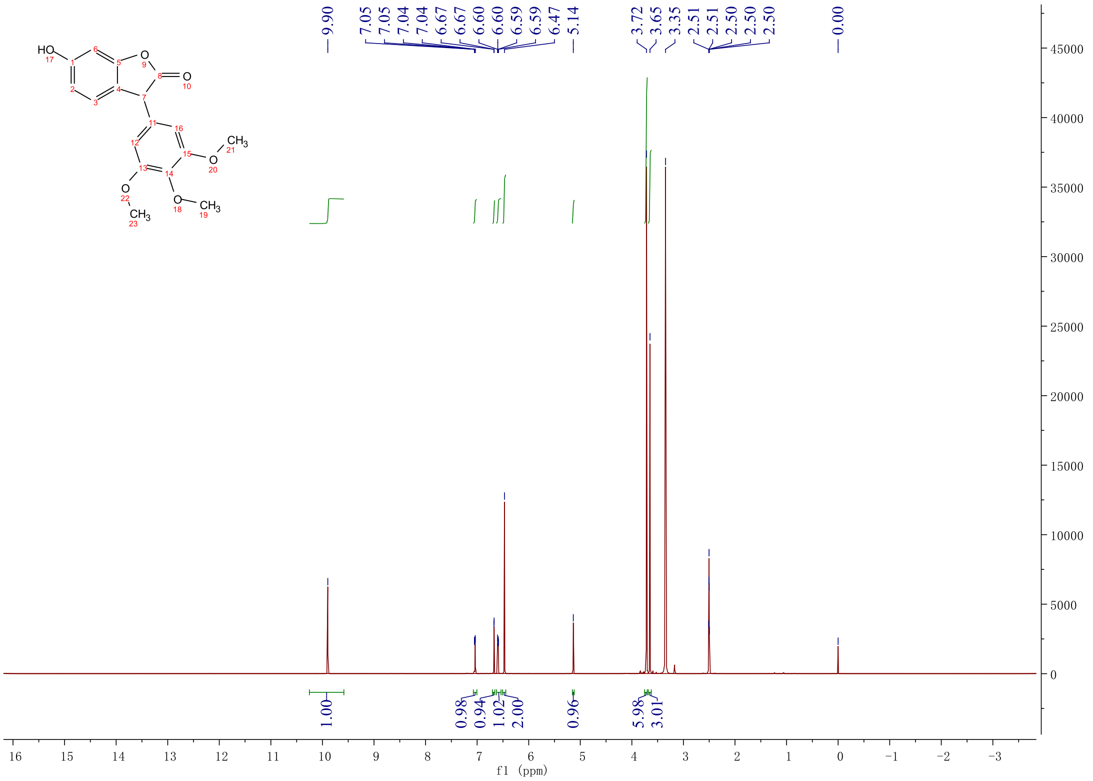

compound **11** ^{13}CNMR

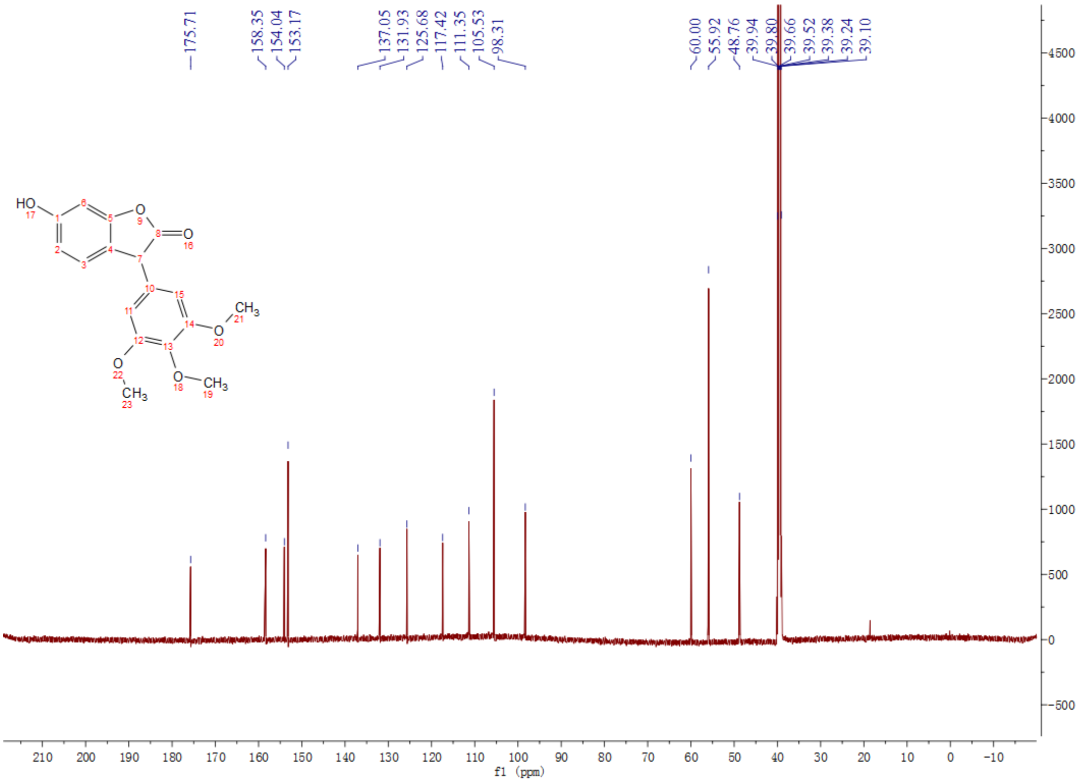

compound **12** ^{1}HNMR

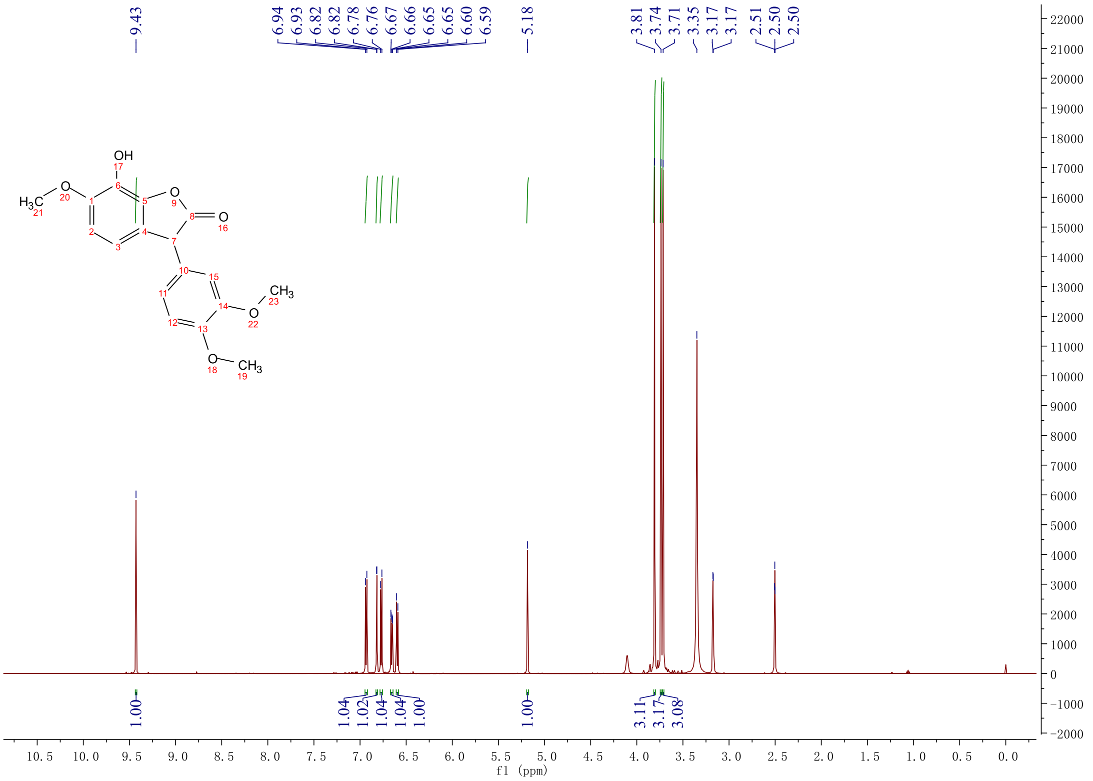

compound **12** ^{13}CNMR

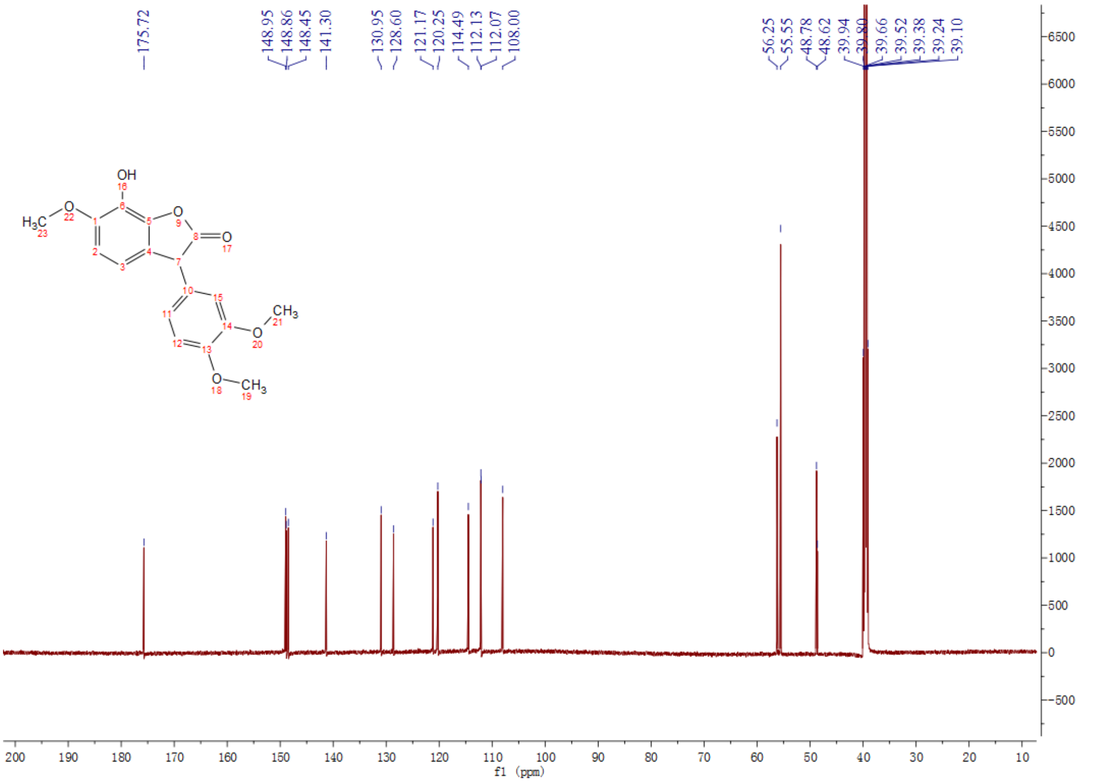

compound **13** ^{1}HNMR

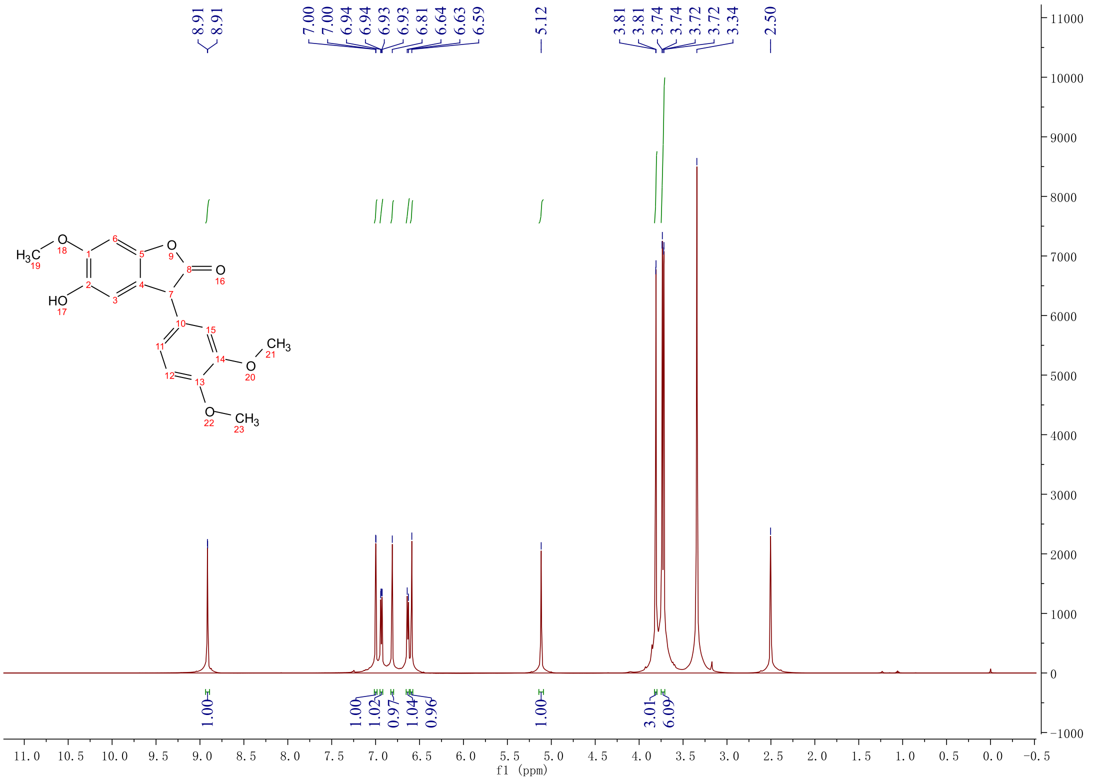

compound **13** ^{13}CNMR

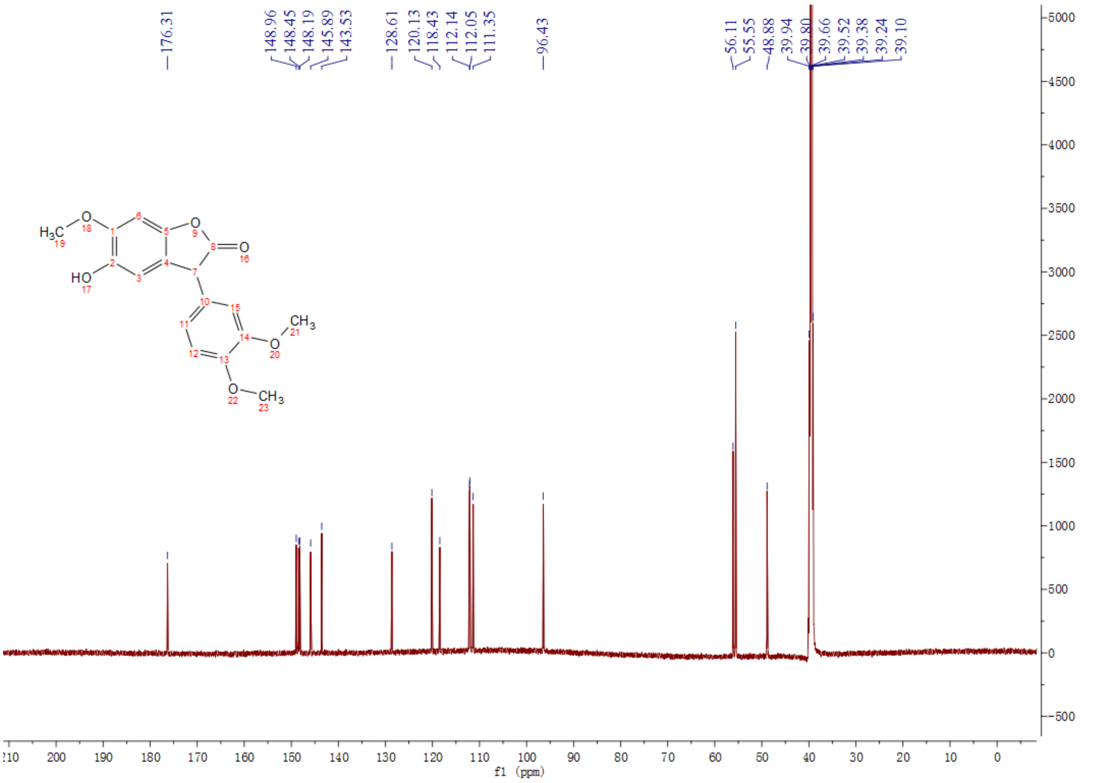

compound **14** ^{1}HNMR

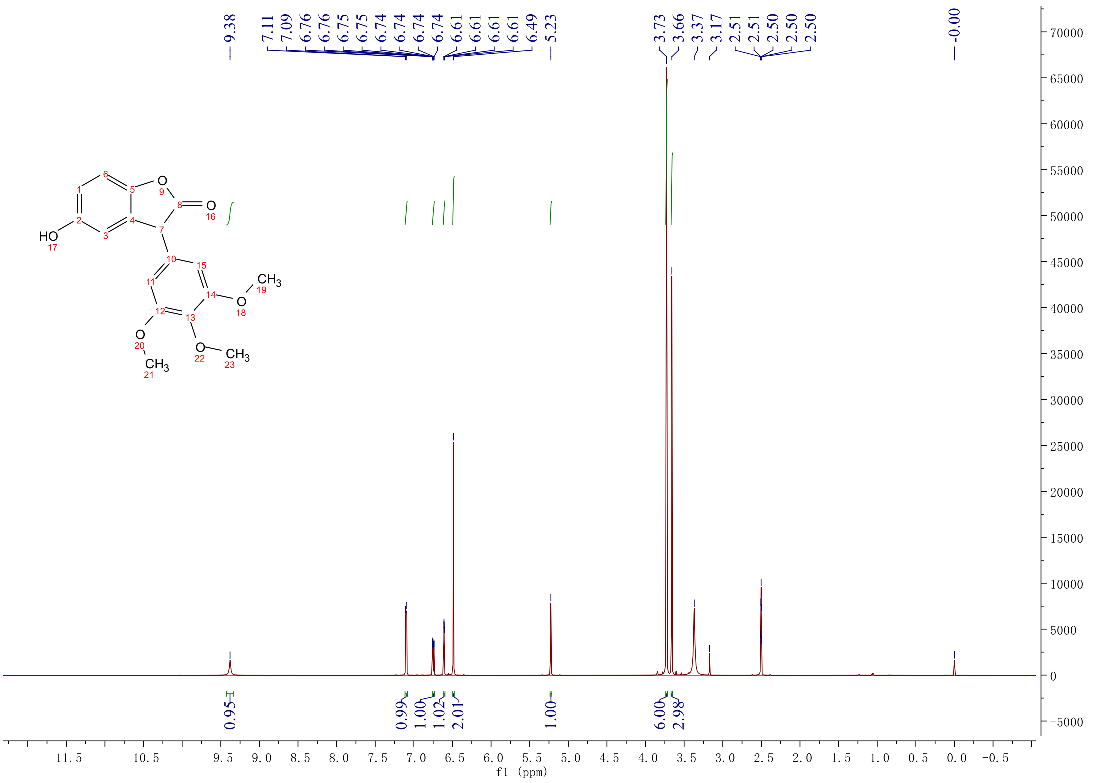

compound **14** ^{13}CNMR

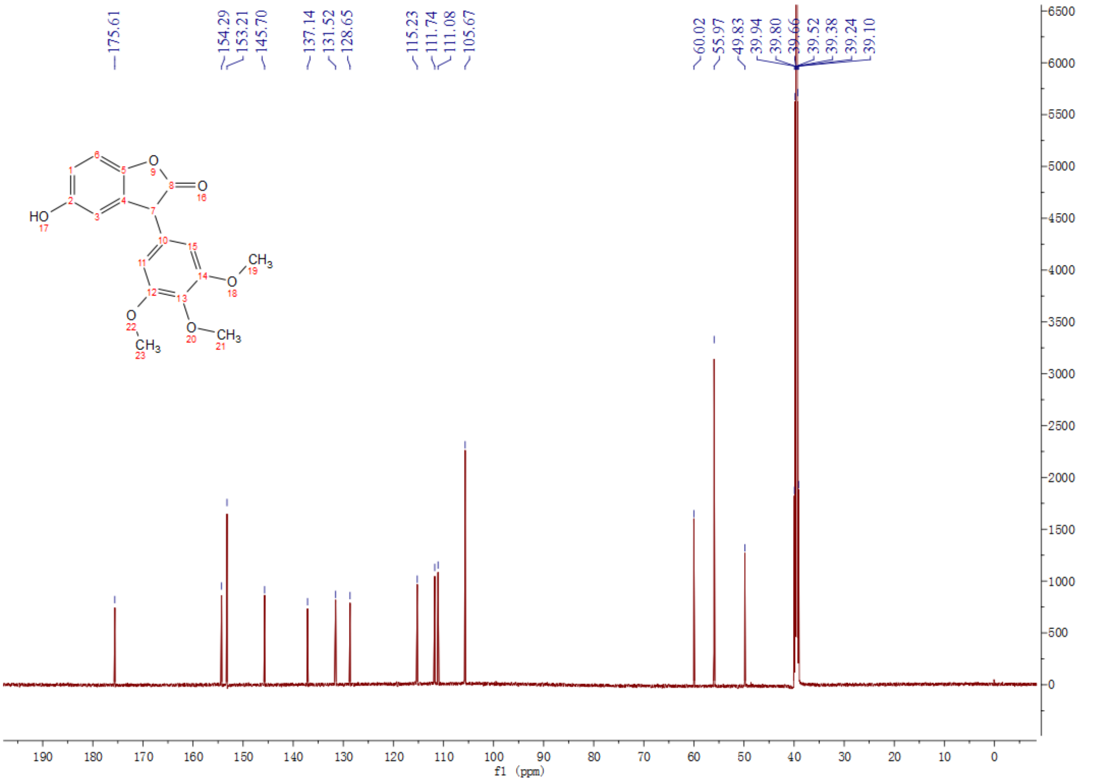

compound **15** ^{1}HNMR

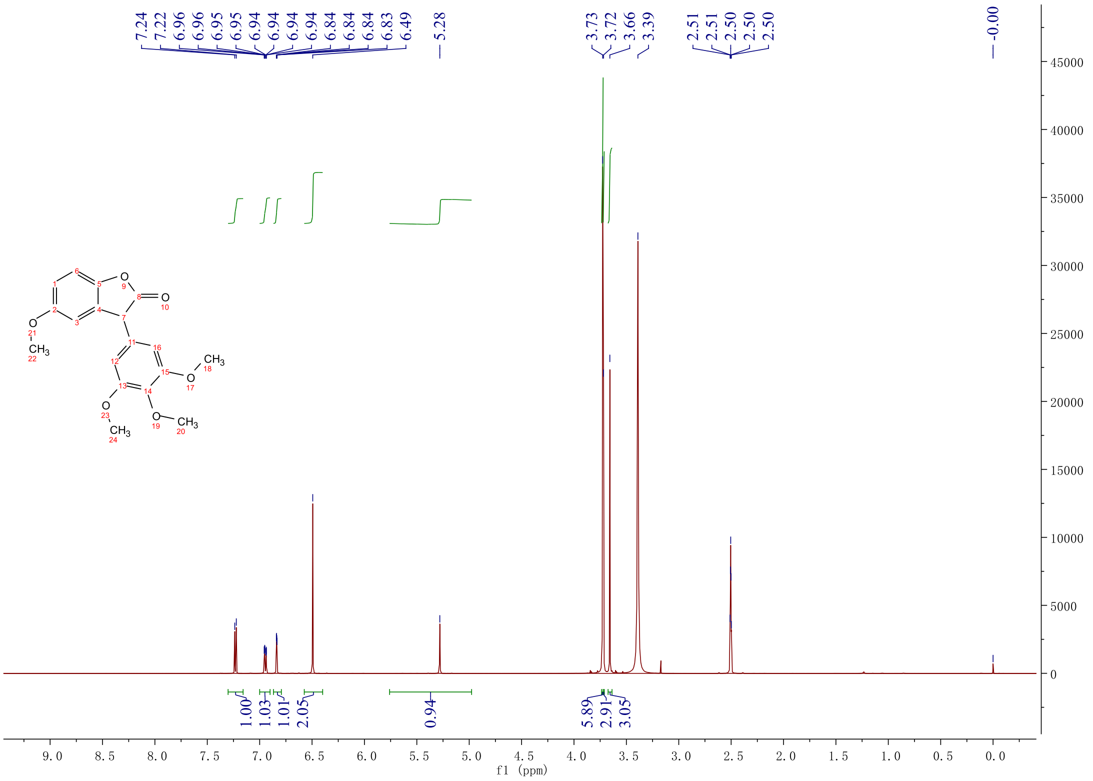

compound **15** ^{13}CNMR

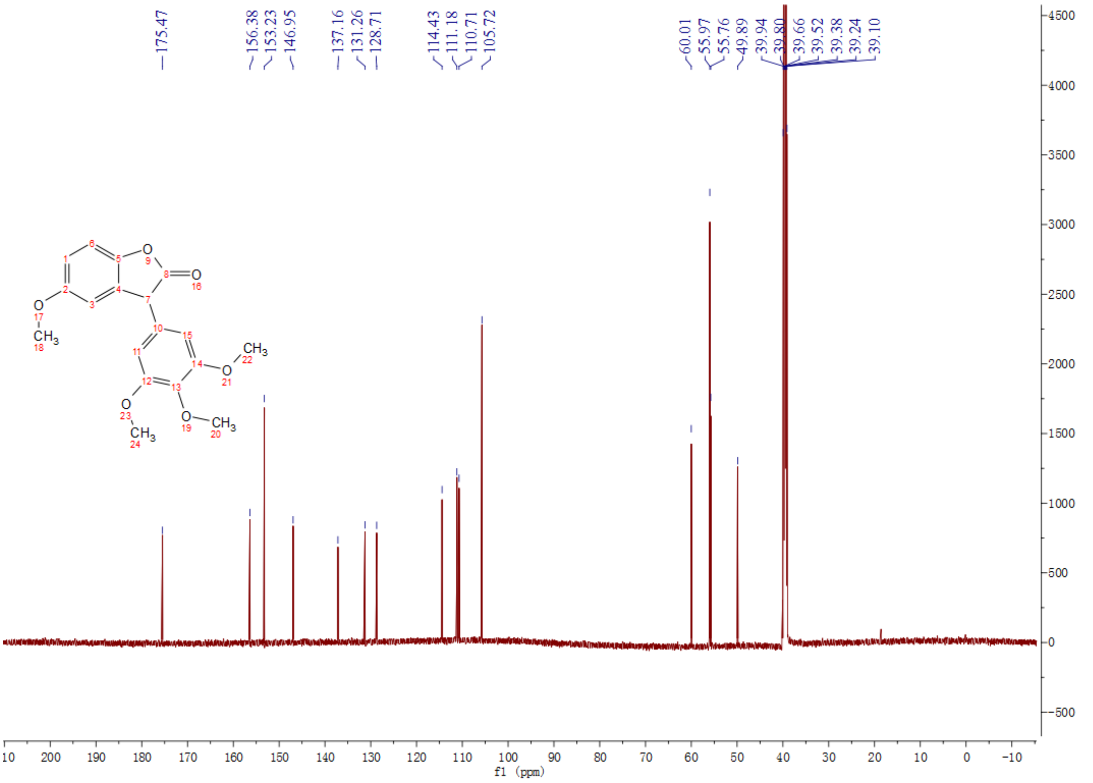

compound **16** ^{1}HNMR

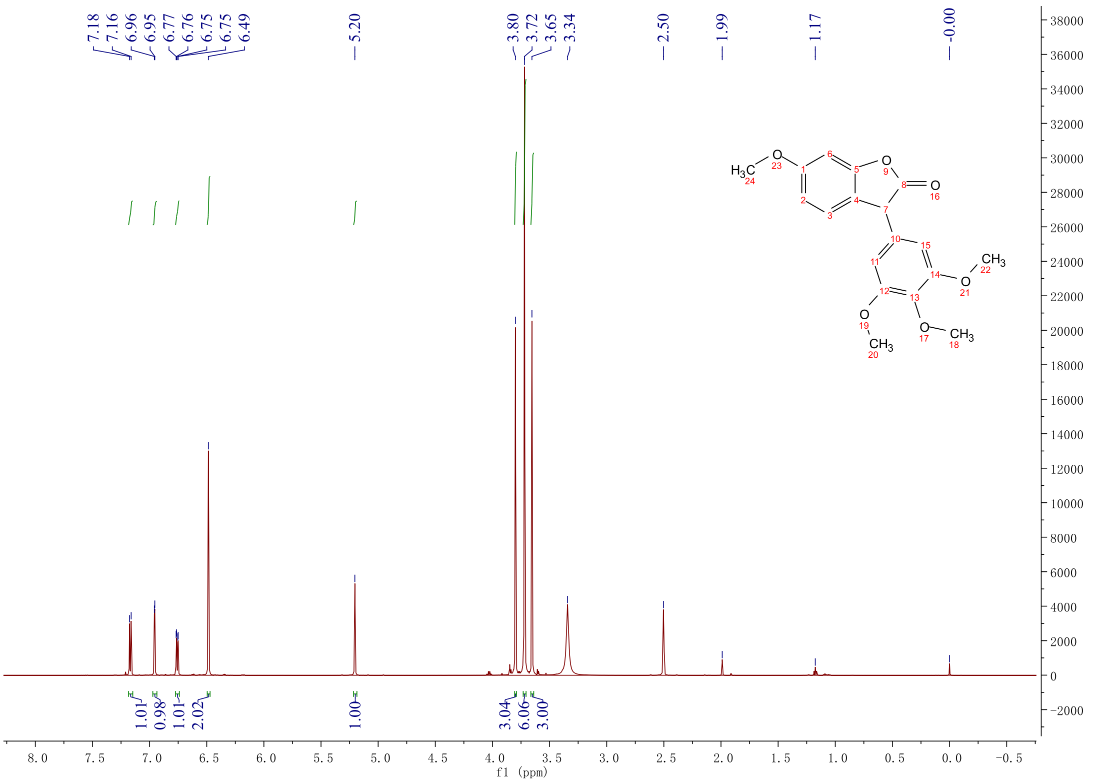

compound **16** ^{13}CNMR

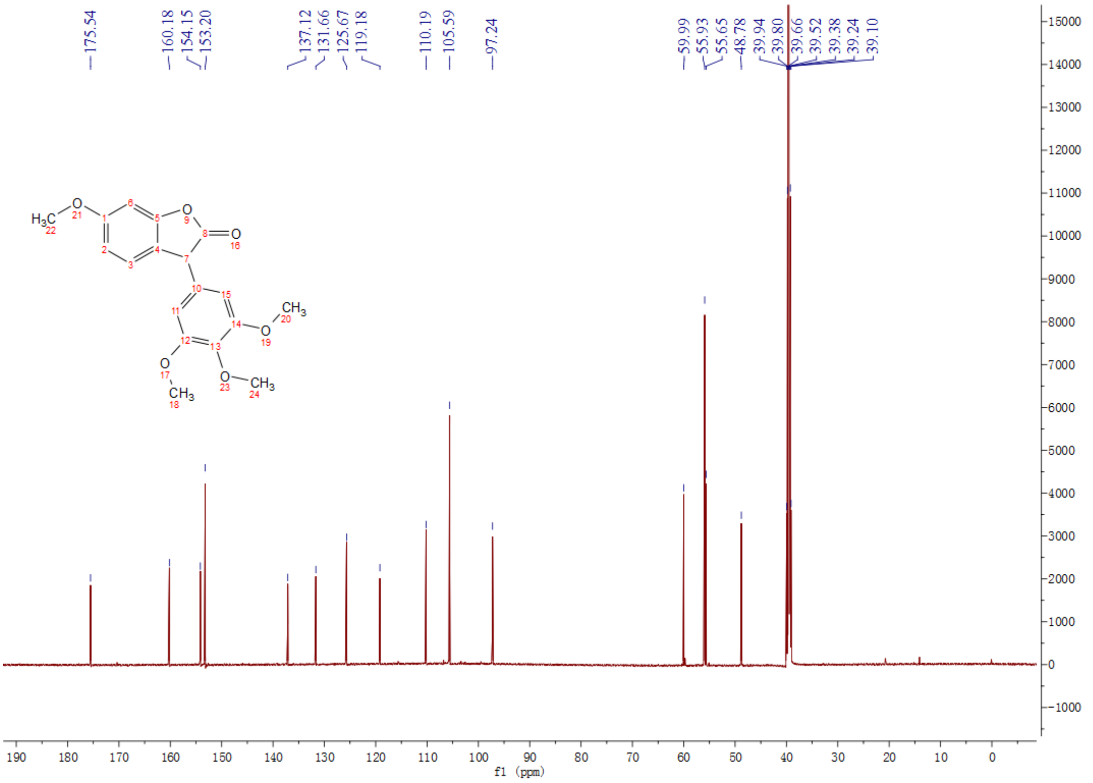

compound **17** ^{1}HNMR

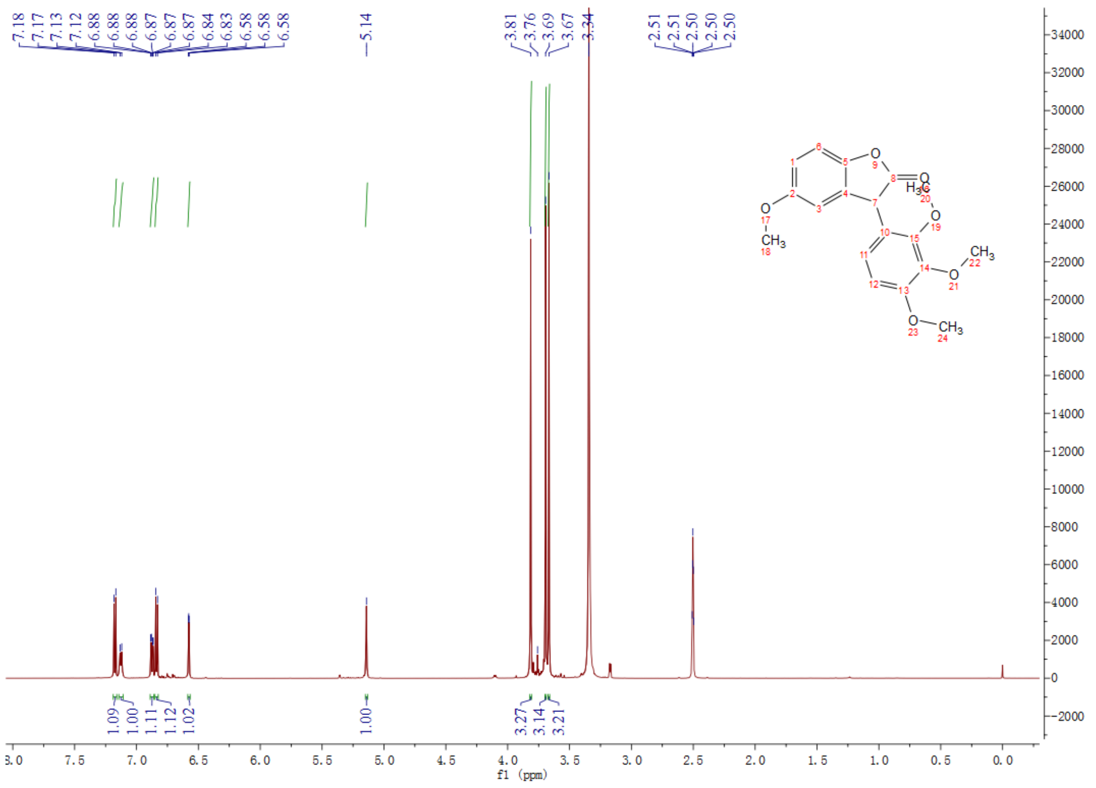

compound **17** ^{13}CNMR

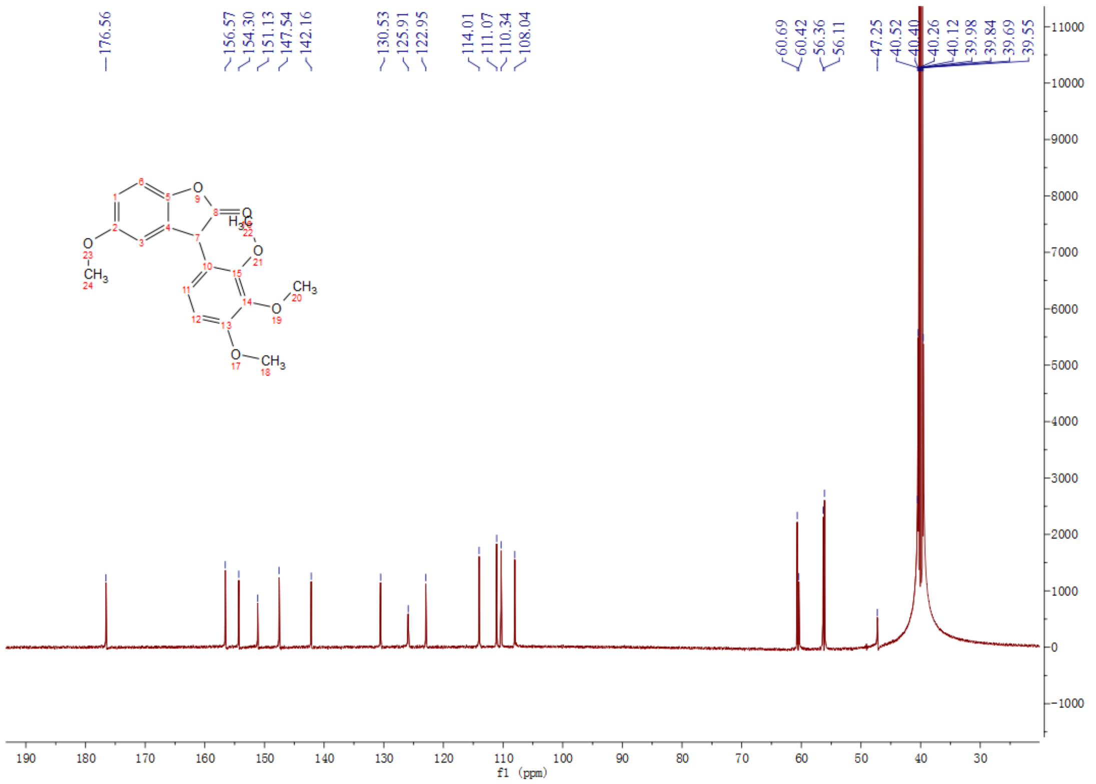

compound **18** ^{1}HNMR

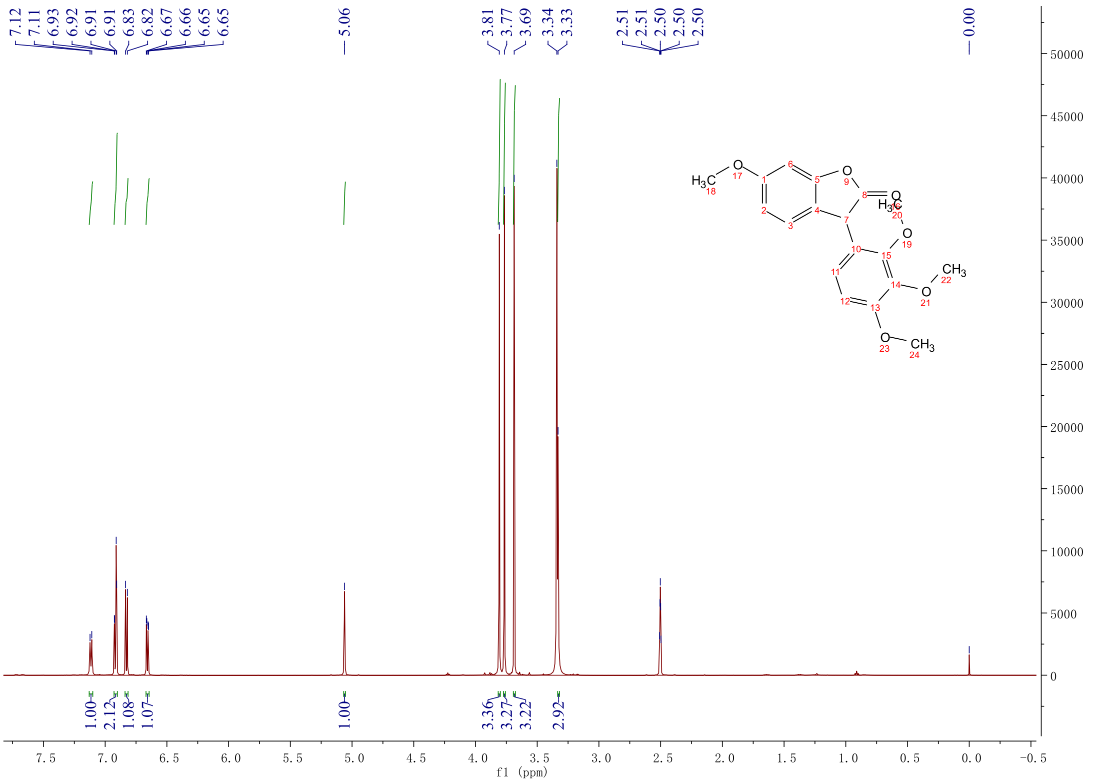

compound **18** ^{13}CNMR

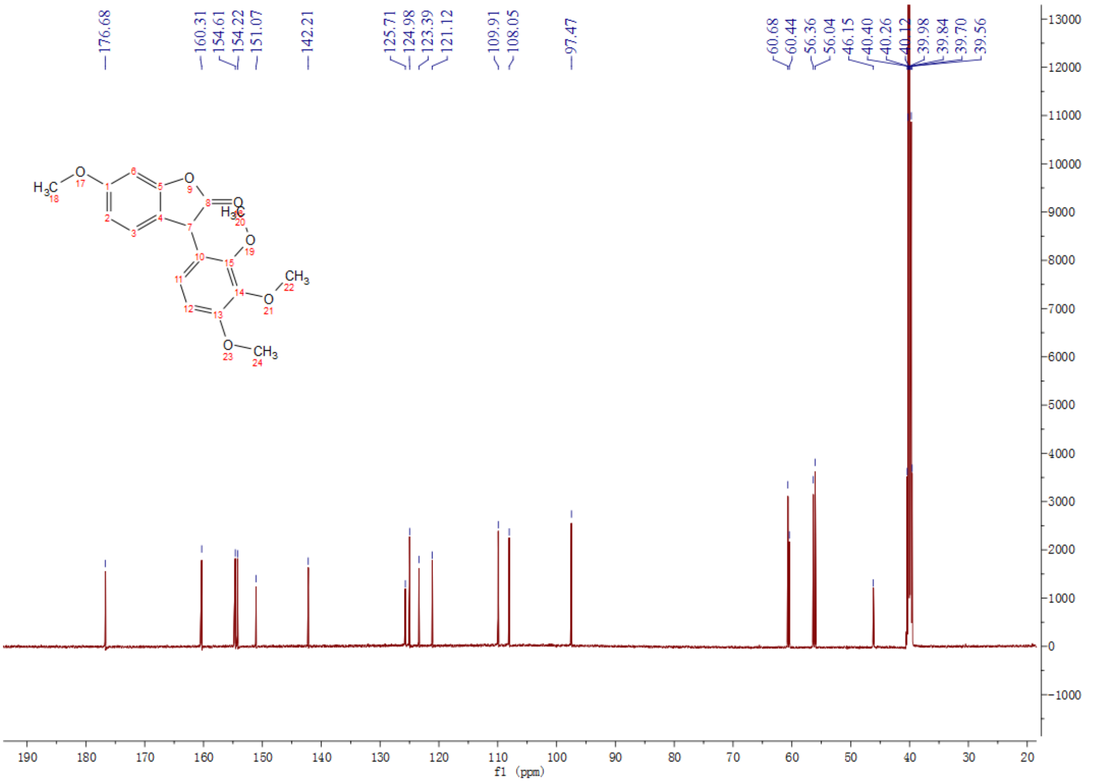

compound **19** ^{1}HNMR

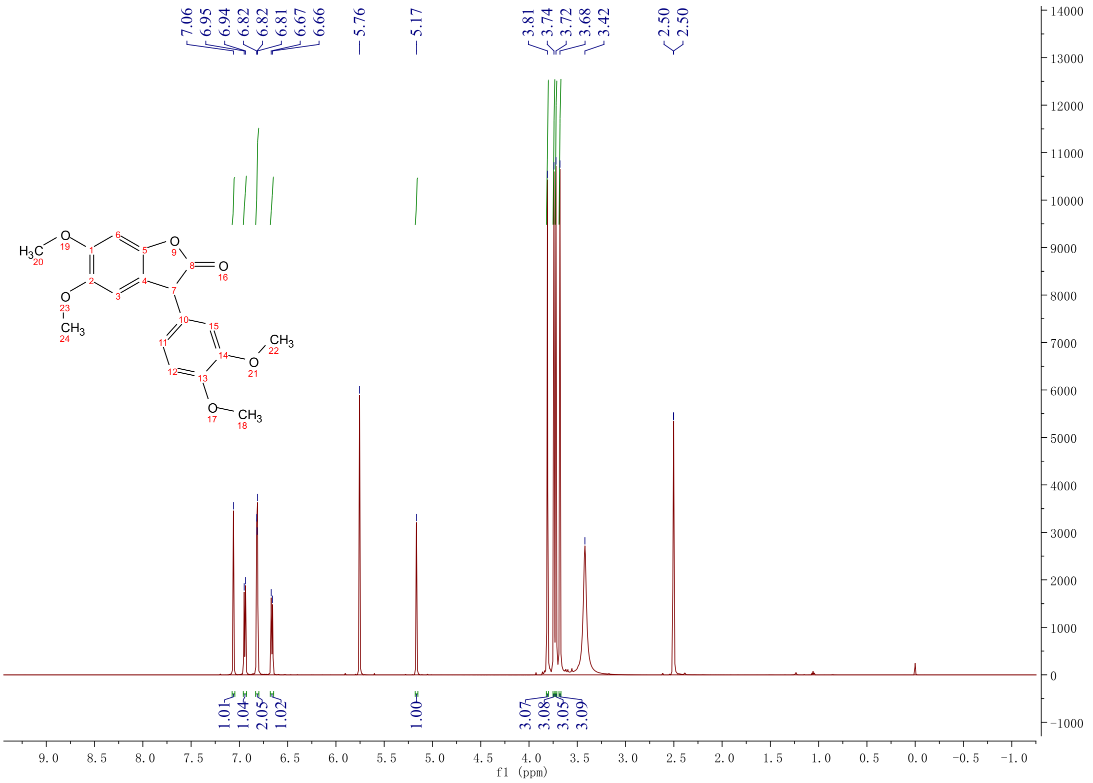

compound **19** ^{13}CNMR

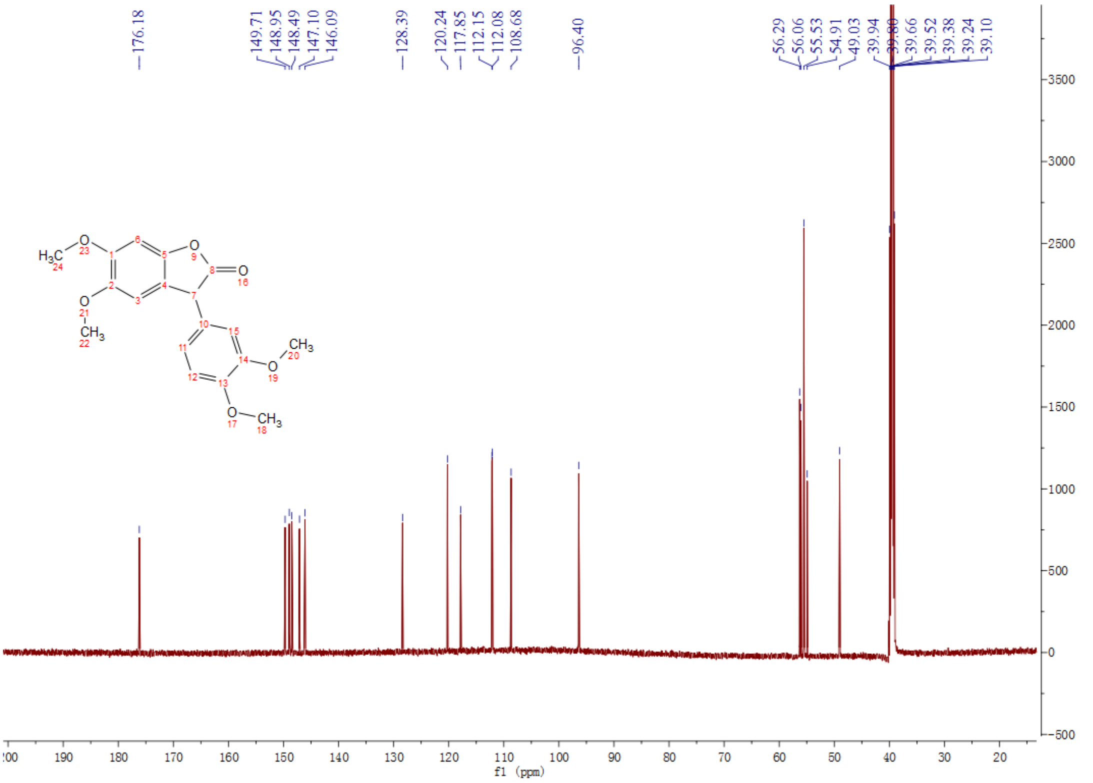

compound **20** ^{1}HNMR

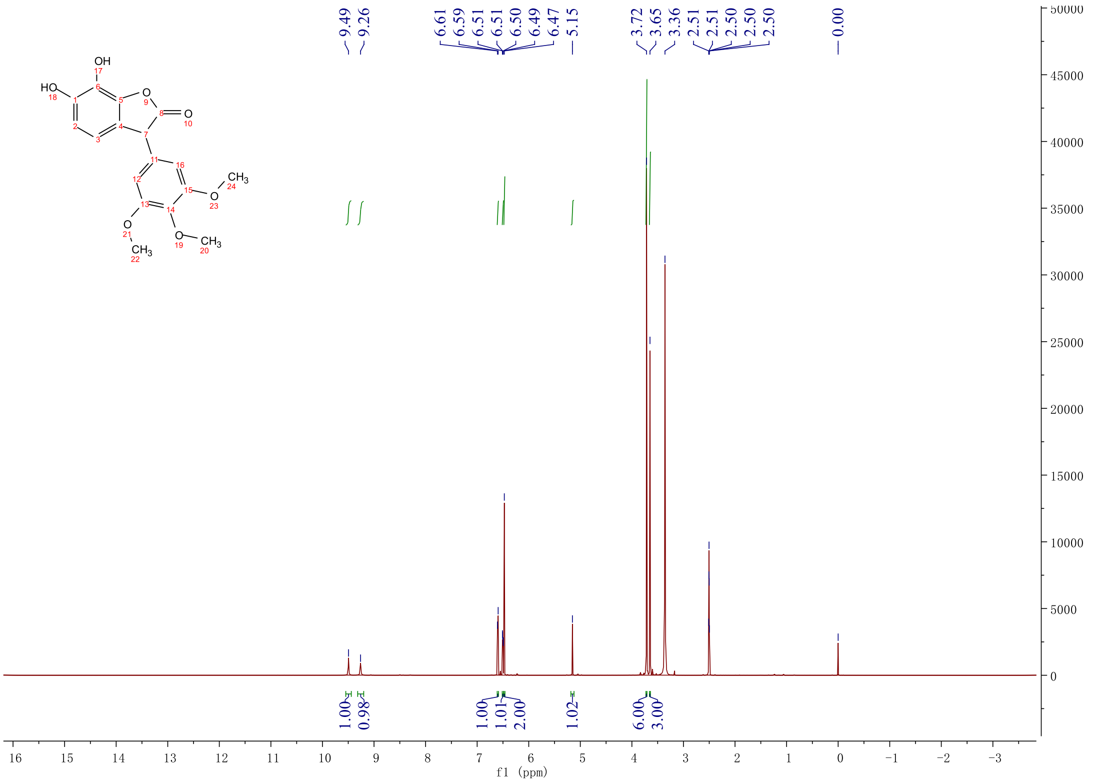

compound **20** ^{13}CNMR

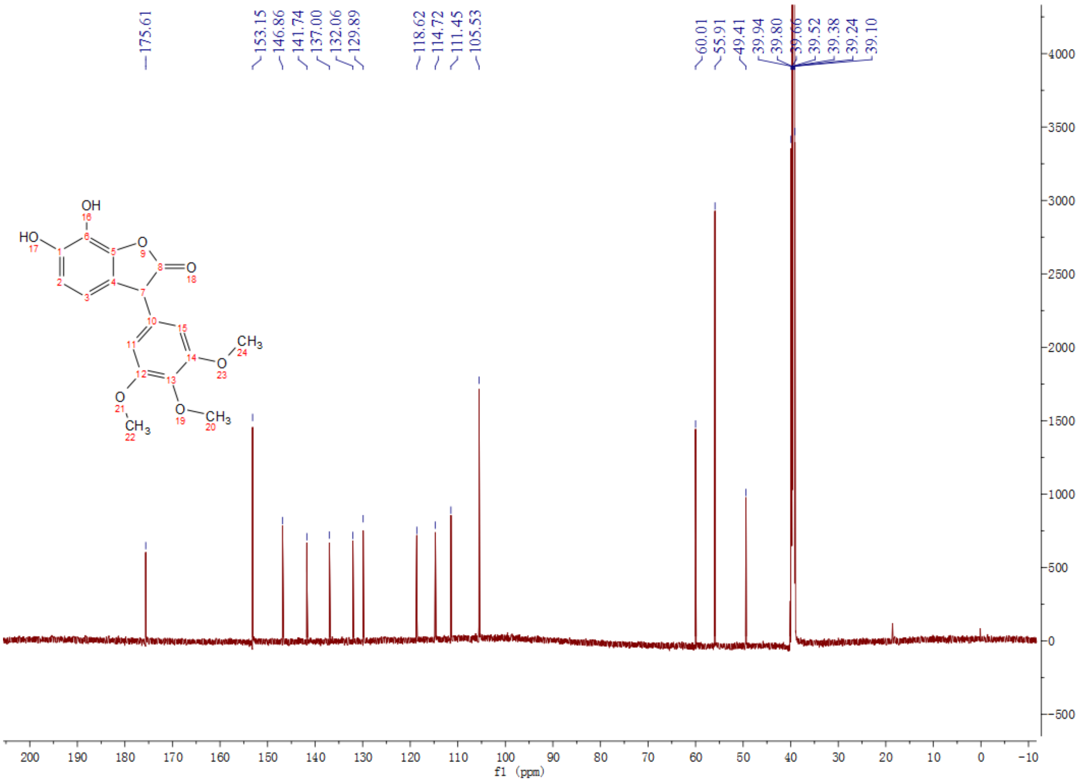

compound **21** ^{1}HNMR

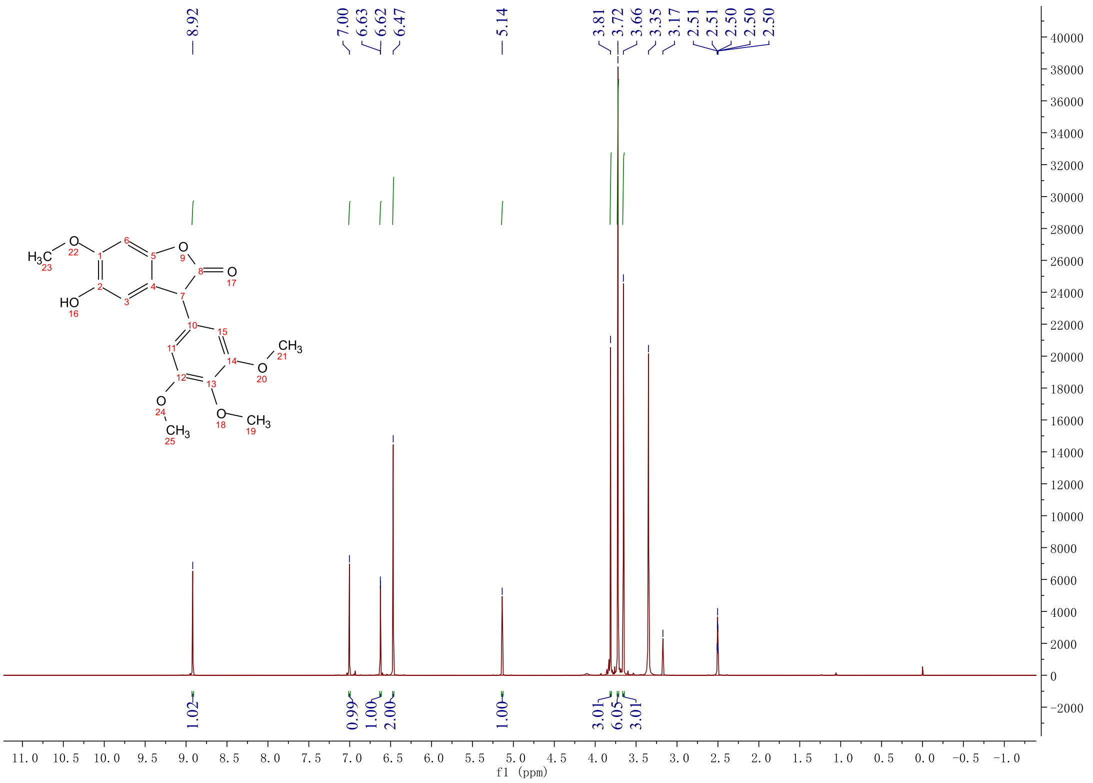

compound **21** ^{13}CNMR

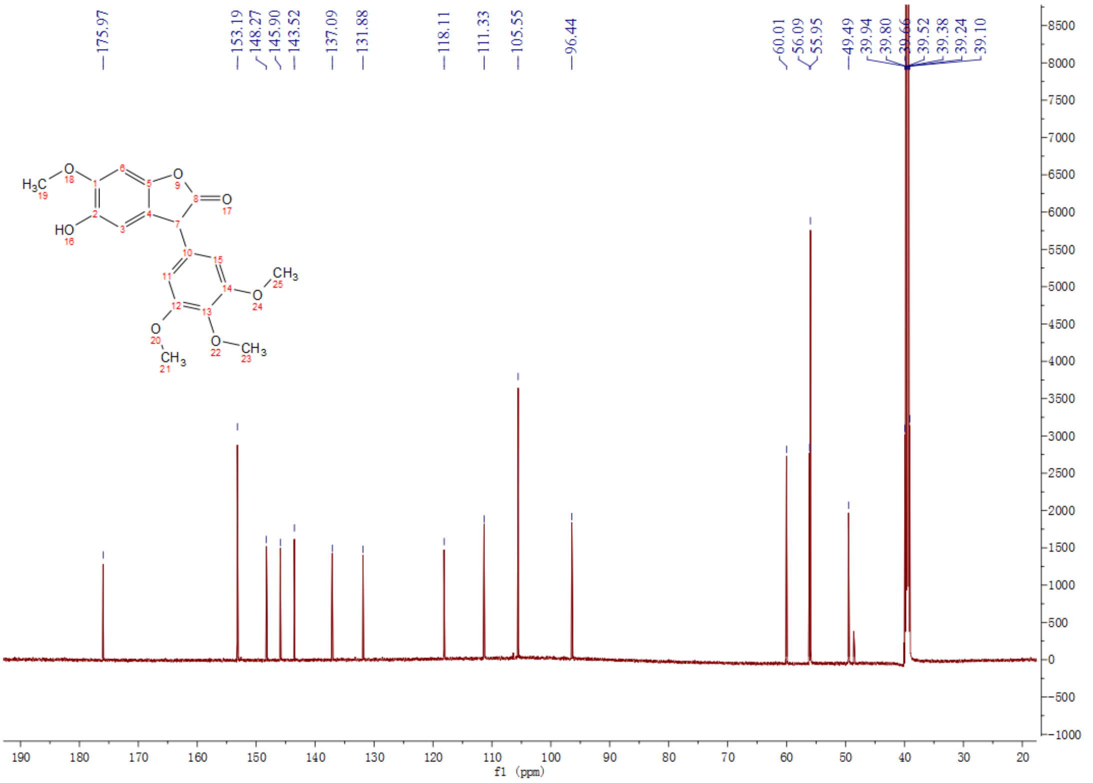

compound **22** ^{1}HNMR

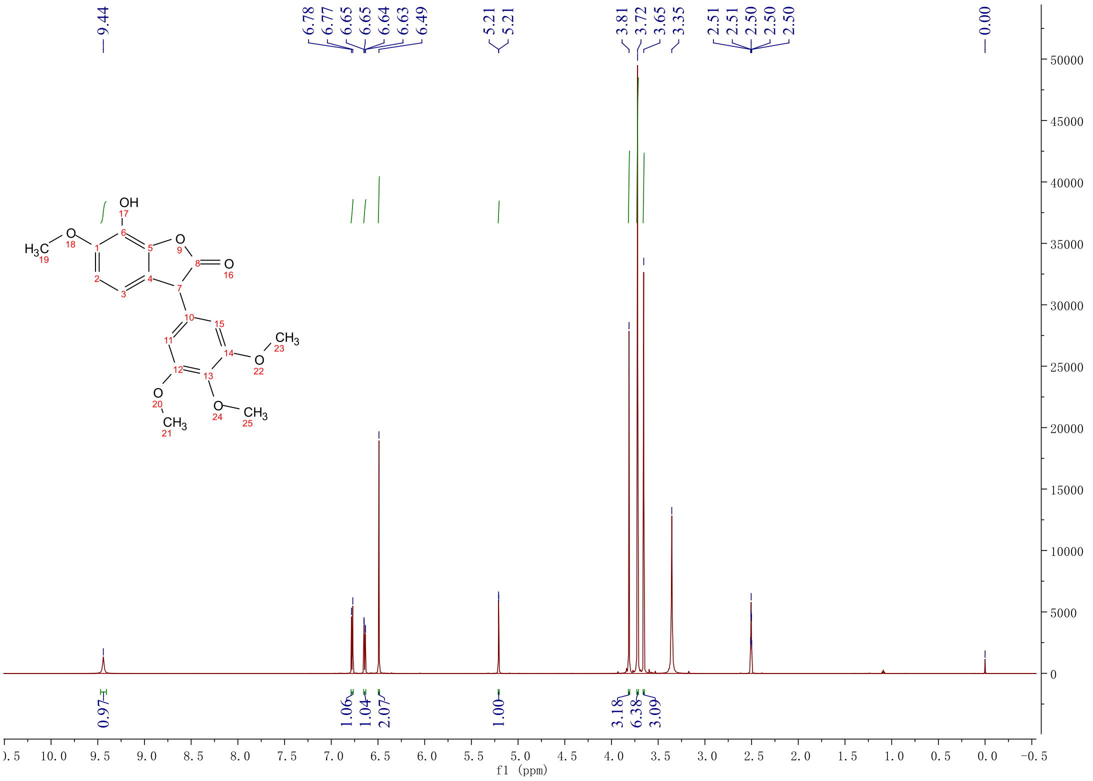

compound **22** ^{13}CNMR

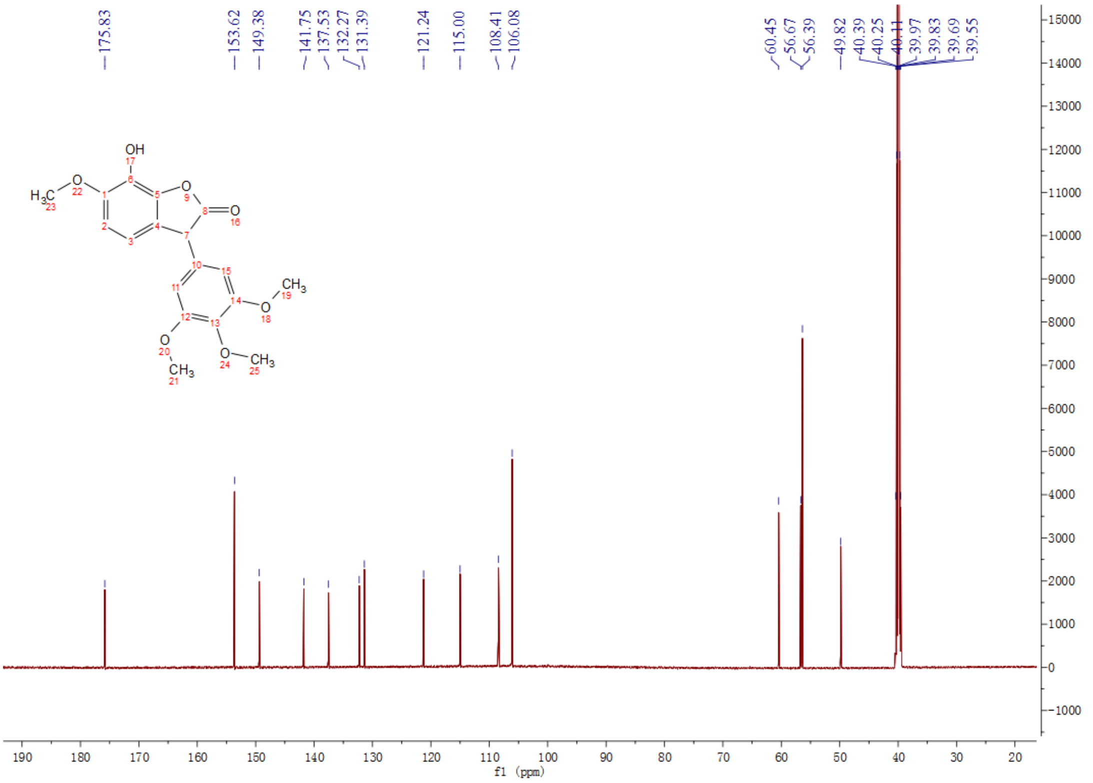

compound **23** ^{1}HNMR

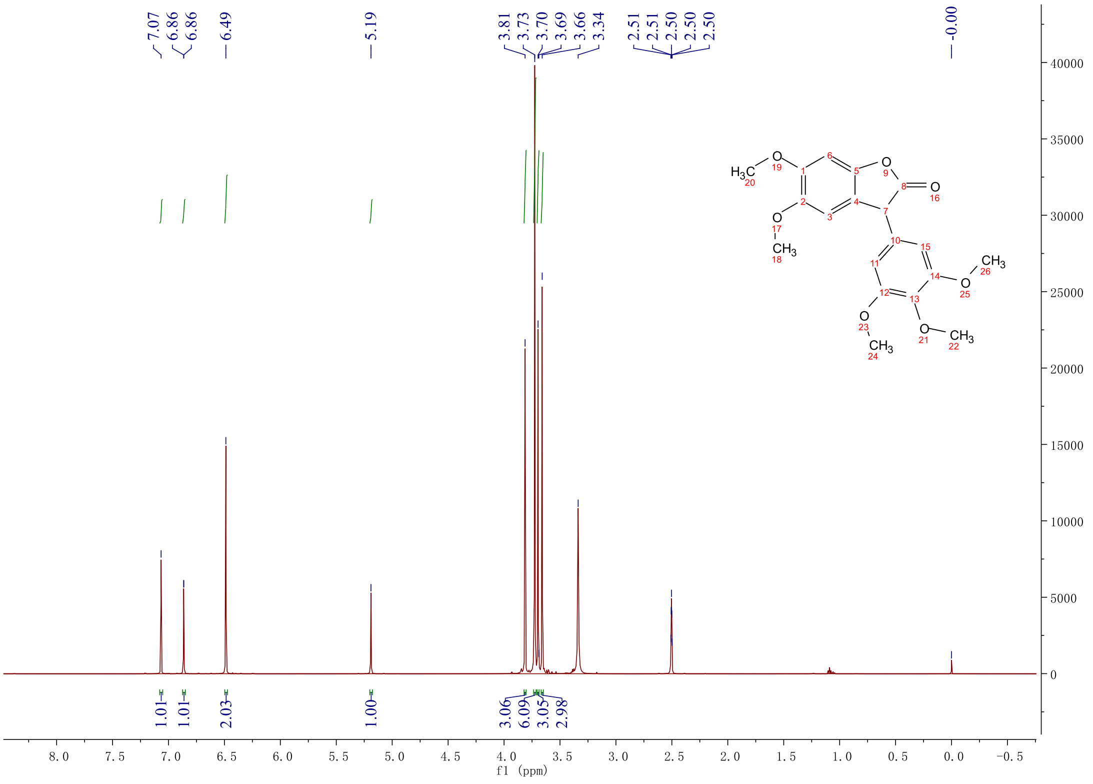

compound **23** ^{13}CNMR

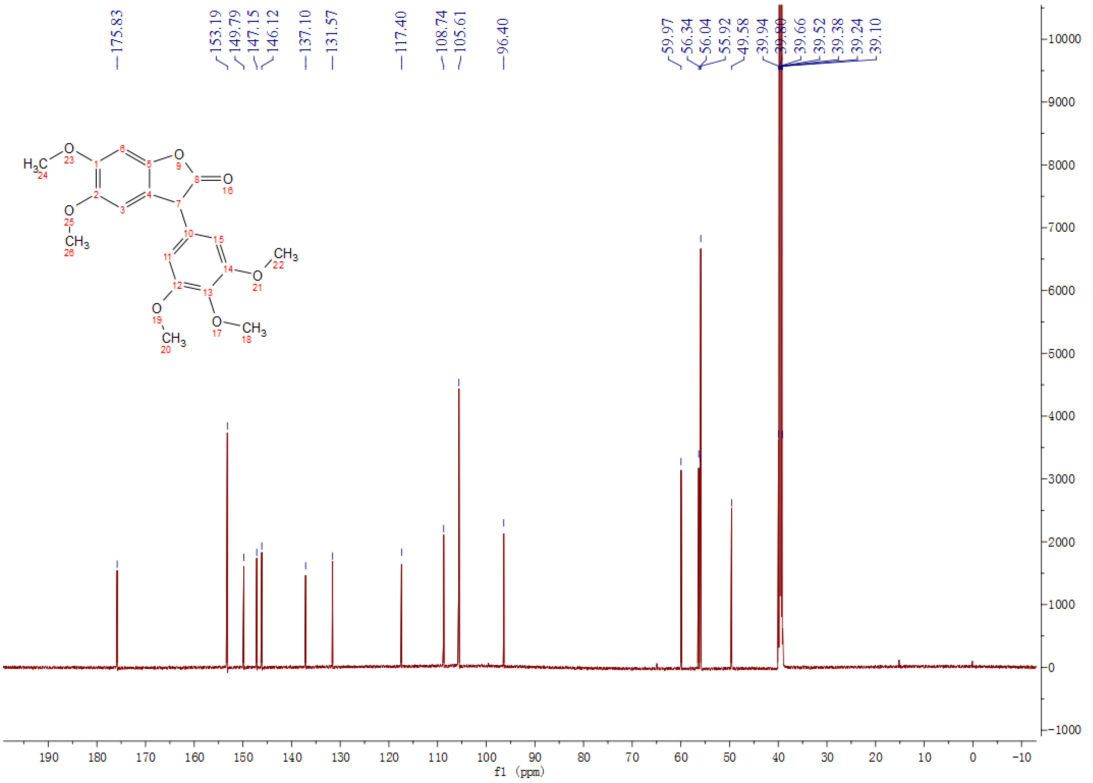

Supplement: Supplemental Material [file IENZ_A_1740694_SM1730.pdf]
